# The Prevalence of Cognitive Impairment in Relapsing-Remitting Multiple Sclerosis: A Systematic Review and Meta-analysis

**DOI:** 10.1007/s11065-024-09640-8

**Published:** 2024-04-08

**Authors:** Wendy Wu, Heather Francis, Abbie Lucien, Tyler-Ann Wheeler, Milena Gandy

**Affiliations:** 1 The School of Psychological Sciences, Australian Hearing Hub, Macquarie University, North Ryde, Sydney, NSW 2109 Australia; 2 Neurology Department, Royal North Shore Hospital, St. Leonards, NSW Australia

**Keywords:** Multiple sclerosis, Cognitive dysfunction, Neuropsychology, Prevalence

## Abstract

It is increasingly recognized that cognitive symptoms are a common sequelae of relapsing-remitting multiple sclerosis and are associated with adverse functional consequences. However, estimates of cognitive impairment (CIm) prevalence vary widely. This study aimed to determine the pooled prevalence of CIm among adults with RRMS and investigate moderators of prevalence rates. Following prospective registration (PROSPERO; CRD42021281815), electronic databases (Embase, Scopus, Medline, and PsycINFO) were searched from inception until March 2023. Eligible studies reported the prevalence of CIm among adults with RRMS, as determined through standardized neuropsychological testing and defined as evidence of reduced performance across at least two cognitive domains (e.g., processing speed, attention) relative to normative samples, healthy controls, or premorbid estimates. The electronic database search yielded 8695 unique records, of which 50 met selection criteria. The pooled prevalence of cognitive impairment was 32.5% (95% confidence interval 29.3–36.0%) across 5859 participants. Mean disease duration and age were significant predictors of cognitive impairment prevalence, with samples with longer disease durations and older age reporting higher prevalence rates. Studies which administered more extensive test batteries also reported significantly higher cognitive impairment prevalence. Approximately one third of adults with RRMS experience clinical levels of CIm. This finding supports the use of routine cognitive testing to enable early detection of CIm, and to identify individuals who may benefit from additional cognitive and functional support during treatment planning.

**Supplementary Information:**

The online version contains supplementary material available at 10.1007/s11065-024-09640-8.

## Introduction

Multiple sclerosis (MS) is a chronic, autoimmune disease which affects approximately 36 per 100,000 people worldwide (Walton et al., 2020). The most common disease course, relapsing-remitting MS (RRMS), constitutes 85% of cases and is marked by distinct episodes of symptom relapse and remission (Leray et al., 2016), while the other disease courses involve progressive worsening of symptoms. Although the most prominent symptoms are in sensorimotor function, increasing emphasis has been placed over recent decades on cognitive effects. Cognitive impairment (CIm) may be a particularly disabling consequence of MS, affecting employment status (Clemens & Langdon, 2018), medication adherence (Bruce et al., 2010), management of finances (Goverover et al., 2019), social functioning (Rao et al., 1991a), and ability to perform activities of daily living (Yazgan et al., 2021). Preservation of cognition is frequently endorsed as a priority among people with MS (Day et al., 2018; Heesen et al., 2008), with one qualitative study highlighting that people with MS were supportive of routine cognitive testing as a means to document an under-addressed, ‘invisible’ symptom, and to advance research in this area (Mortensen et al., 2020).

Despite acceptance that CIm is a potential symptom of MS, estimates of impairment prevalence are equivocal and have not been explored meta-analytically or specifically for people with RRMS. Estimates frequently cited in the literature are broad in range, such as the 40–65% range reported in a seminal literature review (*k* = 11, *n* = 640) by Amato and colleagues in 2006 (Amato et al., 2006), and do not provide a specific overall prevalence estimate. Additionally, the reviewed studies are now many decades old and do not utilize current best practice methods or tests for assessing CIm prevalence (Parsons et al., 1957). Moreover, the samples within studies which sought to estimate CIm prevalence within the MS population are largely unrepresentative as they are small (*n* < 100) or recruited from community samples (McIntosh-Michaelis et al., 1991; Rao et al., 1991a) which may vary in CIm prevalence to clinic-based samples (Amato et al., 2006). Furthermore, these studies did not differentiate between those with RRMS and the progressive courses of MS, which typically involve more severe cognitive dysfunction (Johnen et al., 2017). Thus, the current literature lacks a precise, recent, and representative estimate of the overall prevalence of CIm among people with RRMS. This estimate would be of great interest to neurologists and other health professionals involved in the care of people with RRMS, as many newly diagnosed people with RRMS report concerns about their cognition (Day et al., 2018; Heesen et al., 2008). An updated and more rigorous estimate of CIm prevalence may increase confidence in patient communication regarding the risk of developing CIm. This estimate may also guide service provision by informing resource allocation within MS health care settings toward cognitive health.

The aim of this study was to estimate the pooled prevalence of CIm in RRMS through a systematic review and meta-analysis of the literature. The primary outcome of interest was CIm prevalence as determined through standardized neuropsychological testing, where at least two cognitive domains (e.g., attention, memory, executive functioning) must be reduced to meet criteria for CIm. This is not only considered best practice (Hancock et al., 2022) but also allows exclusion of studies which only examine one cognitive domain, given that domains are differentially affected in MS (Prakash et al., 2008) and single-domain studies are thus likely to misrepresent the overall CIm prevalence. This restriction was also adopted to increase the precision of the prevalence estimate, particularly as previous meta-analyses estimating CIm prevalence in other conditions have found the high variability of definitions across studies to be a barrier to interpretation of their findings (Rayes et al., 2018; Sexton et al., 2019; Yohannes et al., 2017). However, other definitional aspects of CIm, such as the standards deemed to indicate impaired test performance, also have the potential to impact estimates of prevalence. Thus, a secondary aim of this study was to review the definitions of CIm used and examine whether this, in addition to clinical, demographic, and methodological variables, moderates reported prevalence rates.

## Method

### Registration

This study was conducted according to the Preferred Reporting Items for Systematic Review and Meta-Analyses (PRISMA) checklist. The protocol was prospectively published on PROSPERO (CRD42021281815).

### Eligibility Criteria

Studies were included if they determined the prevalence of CIm using standardized neuropsychological measures among adults (at least 18 years) diagnosed with RRMS, and reported the proportion of participants who were impaired in two or more cognitive domains. Also eligible were studies which used more than one test to measure a single cognitive domain, and defined CIm as two tests in the impaired range spread across at least two different domains.

Exclusion criteria were studies which (1) only used a cognitive screening measure rather than a neuropsychological test battery, e.g., Montreal Cognitive Assessment (MoCA) or Mini-Mental State Exam (MMSE), given reports that screens have limited sensitivity for detecting CIm in MS (Portaccio et al., 2009a, b; Sehanovic et al., 2020); (2) used self-report rating scales to determine CIm; (3) only required impairment in one cognitive domain for CIm and did not report proportion of participants impaired in two or more domains; (4) aggregated performances over multiple cognitive domains to a single index, as it was possible for individuals with an impairment in only one domain to meet CIm criteria under these conditions; (5) recruited participants with various disease courses and did not report the CIm prevalence for those with RRMS; (6) included participants with pediatric-onset MS; and (7) gauged participants’ cognitive status to determine their eligibility to participate (e.g., required a minimum score on a cognitive screen, only recruited people with cognitive complaints).

Where the same cohort was examined across multiple timepoints, only the publication associated with data collected from the first timepoint was included. Additional data were requested from corresponding authors where relevant.

### Search Strategy and Study Selection

Embase, Scopus, Medline, and PsycINFO were searched from inception to March 2023, with no restriction to sample size or the year or country of publication. The search strategy (Fig. 1 of the Supplement) combined keywords and Medical Subject Heading terms related to (1) multiple sclerosis; (2) CIm; and (3) neuropsychological testing. Duplicates were removed using an automated EndNote function.

One reviewer (W. W.) screened all titles and abstracts using the application Rayyan (Ouzzani et al., 2016) and a random 20% was independently assessed by a second reviewer (T. W.) to verify the accuracy and consistency of the screening process. Full-text articles were independently assessed by reviewers (W. W. and T. W.) using a standardized eligibility spreadsheet, with excellent agreement (*κ* = .84). Studies published in non-English languages were reviewed following translation with Google Translate, which was effective for translating articles to review information relevant to the current study. Discrepancies were resolved by consensus, including with a third reviewer (H. F.).

### Data Extraction

A standardized data extraction spreadsheet was used by two independent reviewers (W. W. and T. W.). The primary outcome extracted was the number of participants with RRMS deemed to have CIm. Where multiple prevalence rates were reported according to different CIm criteria, the rate corresponding with the authors’ primary definition of CIm was extracted. Other extracted data included study characteristics (country, recruitment setting, RRMS sample size), demographic and clinical variables (age, gender, disease duration, Expanded Disability Status Scale [EDSS]), and details about the measurement and definition of CIm (neuropsychological tests, cut-off used to indicate test impairment, number of impaired domains required for CIm, and whether healthy controls, normative samples, or premorbid estimates were used as the comparison group).

Study quality was independently assessed by two reviewers (W. W. and T. W.) according to the modified version of the Quality Assessment of Diagnostic Accuracy Studies (QUADAS) tool (Broen et al., 2012; Broen et al., 2016; Leboeuf-Yde & Lauritsen, 1995), available in Table 1 of the Supplement. The QUADAS has been recommended by the Cochrane Collaboration (Whiting et al., 2011), and was modified by Broen and colleagues (2012) according to criteria developed by Leboeuf-Yde and Lauritsen (1995) and Walker and colleagues (2000) to increase applicability for prevalence studies. It includes 10 criteria regarding participant representativeness, data quality, description of method and results, and definition of prevalence. Points are allocated for each criterion, which are then summed into a model-free count score without estimation of a statistical model (Scherer & Emslander, 2024). A maximum score of 19 points can be earned, with a score of 14 or higher indicating acceptable quality (Broen et al., 2012).

### Statistical Analyses

Comprehensive Meta-Analysis (CMA) version 3.0 (Borenstein et al., 2013) was used to conduct analyses and generate plots. Following logit transformations of the prevalence rates, the pooled prevalence of CIm was estimated using a random-effect model with 95% confidence intervals (CIs). The logit-transformed prevalence rates were back-transformed into proportions to aid interpretability and ease of reporting. Between-study heterogeneity was assessed using the *I*^2^ statistic, with values above 40% considered to indicate moderate heterogeneity and values above 60% considered substantial (Deeks et al., 2019).

Meta-regression and subgroup analyses were conducted to determine moderators of CIm prevalence. Continuous variables of interest were year of publication, RRMS sample size, mean age, mean disease duration, proportion of female participants, and number of neuropsychological tests administered. Categorical variables were aspects of the definition of CIm used, including the cut-off used to determine impaired test performance (1.5 standard deviations [SDs], 1.67 SDs, or 2 SDs below the comparator’s mean), number of impaired tests required (2 or >2), and comparison group (normative values or healthy controls). As no studies used premorbid estimates, we were unable to include this as a level within subgroup analyses. Many studies reported EDSS using medians rather than means. We extracted whichever was reported and recoded this to a categorical variable. As no studies reported EDSS scores corresponding to severe neurological disability and only four studies reported scores in the moderate range, those which reported EDSS below 2.0 (i.e., no disability) were recoded as lower EDSS and those 2.0 or greater were recoded as higher EDSS (Kurtzke, 1983). Study quality was also recoded to a categorical variable as it was inappropriate for meta-regression given the modified QUADAS yield model-free sum scores (Scherer & Emslander, 2024) and due to the restricted scores obtained. Majority of studies earned the same score of 12/20, with only one study meeting the minimum acceptable score of 14/20. Hence, studies with scores below 12 were recoded as lower quality and scores at or above 12 were recoded as higher quality, and were subject to subgroup analyses to identify whether the studies which earned a score lower than the mode of 12 reported different prevalence rates to the majority.

Risk of publication bias was assessed using a funnel plot and Egger’s test. Funnel plots which appear asymmetric and significant Egger’s tests are suggestive of publication bias. The trim-and-fill procedure was used to determine whether removal of any studies would improve funnel plot symmetry (Duval & Tweedie, 2000).

## Results

**Fig. 1:**
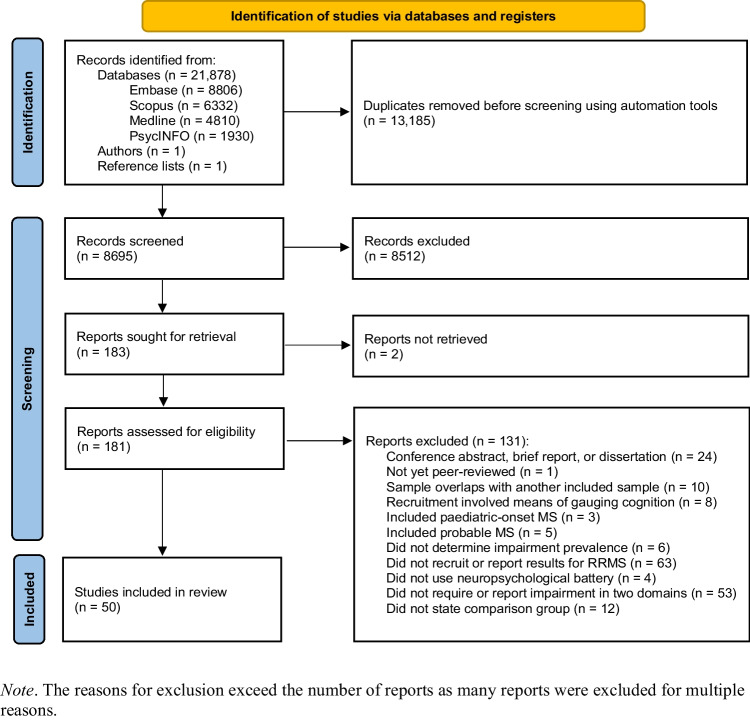
Flow chart of the selection process for studies

The search yielded 21,878 articles, of which 13,185 were duplicates. Two additional articles were sourced from correspondence with authors and reference lists. Of the 8695 titles and abstracts screened for eligibility, 183 were sought for full-text review. Fifty studies, which included data from 5859 people with RRMS, met the inclusion criteria and were included in the analyses (reference list in Fig. 2 of the Supplement). Details of the selection process are available in Fig. 1.

### Study Characteristics

Characteristics of included studies are available in Table 1. Year of publication ranged from 2004 to 2023. All studies which reported the location where participants were recruited did so from a clinical setting (*n* = 43; 86%), most commonly a MS center (*n* = 32; 64%), with one study utilizing both clinical and community sampling. Thirty-one studies (62%) solely recruited participants with RRMS, and the remainder recruited mixed samples with different disease courses. Some mixed sample studies did not report demographic data for the participants with RRMS; these studies were included in the overall CIm prevalence estimate but were unable to be included in the moderator analyses if they did not report the relevant moderator.

**Table 1:** Characteristics of included studies

**Study/country**	***N***	**Mean age (SD)**	**Female (%)**	**Mean EDSS (SD)**	**Mean DD, years (SD)**	**Number; neuropsychological tests administered**	**CIm definition**	**Reference group**	**Rate of Cim (95% CI)**	**Study quality**
Akbar et al. (2010)/Canada	49	NR for RRMS	NR for RRMS	NR for RRMS	NR for RRMS	4; NSBMS (COWAT, PASAT, 7/24 SpaRT, SRT)	<1.67 SDs, 2 tests	Norms	12.2% (5.6–24.7%)	12
Altieri et al. (2021)/Italy	82	45.8 (11.0)	56 (68.3)	2.8 (1.8)	3.9 (2.0)	10; BICAMS (BVMT-R, CVLT-II, SDMT), AAT, Phonemic Fluency, RCFT, Semantic Fluency, Stroop, TMT, WTET	<2 SDs, 2 domains	Norms	23.2% (15.3–33.5%)	12
Amato et al. (2004)/Italy	41	35.1 (8.6)	30 (73.2)	1.5 (0.6)	4.0 (2.8)	5; BRB-N (PASAT, SDMT, 10/36 SpaRT, SRT, WLG)	<2 SDs, 1 test^*^	Norms	22.0% (11.8–37.1%)	12
Amato et al. (2008)/Italy	47	46.4 (8.4)	32 (68.1)	1.3 (0.9)	22.5 (6.0)	6; BRB-N (PASAT, SDMT, 10/36 SpaRT, SRT, WLG), Stroop	<2 SDs, 3 tests	Norms	23.4% (13.5–37.5%)	12
Amato et al. (2010)/Italy	49	36.9 (8.9)	38 (77.6)	1.7 (0.7)	2.9 (1.7)	6; BRB-N (PASAT, SDMT, 10/36 SpaRT, SRT, WLG), Stroop	<1.5 SDs, 2 tests	HCs (*n* = 56)	30.6% (19.4–44.7%)	12
Amato et al. (2012)/Italy	26	34.9 (7.8)	18 (69.2)	1.6 (1.0)	8.7 (7.1)	6; BRB-N (PASAT, SDMT, 10/36 SpaRT, SRT, WLG), Stroop	<1.67 SDs, 2 tests	Norms	34.6% (19.1–54.3%)	12
Bisecco et al. (2015)/Italy	52	40.3 (8.5)	33 (63.5)	2.0 (Mdn)	8.4 (NR)	6; BRB-N (PASAT, SDMT, 10/36 SpaRT, SRT, WLG), WCST	<2 SDs, 2 tests	Norms	42.3% (29.7–56.0%)	12
Caceres et al. (2014)/multi-SA	110	36.6 (10.6)	74 (67.3)	2.1 (1.3)	Inclusion criteria <5	5; BRB-N (PASAT, SDMT, 10/36 SpaRT, SRT, WLG)	<1.67 SDs, 2 domains	HCs (*n* = 34)	34.5% (26.3–43.9%)	12
Carotenuto et al. (2022)/multi-EU	704	40.7 (10.7)	476 (67.6)	2.0 (Mdn)	9 (Mdn)	5; BRB-N (PASAT, SDMT, 10/36 SpaRT, SRT, WLG)	<1.5 SDs, 2 tests	Norms	36.2% (32.8–39.8%)	12
Conti et al. (2021)/Italy	198	NR for RRMS	NR for RRMS	NR for RRMS	NR for RRMS	5; BRB-N (PASAT, SDMT, 10/36 SpaRT, SRT, WLG)	<1.5 SDs, 2 tests	Norms	20.7% (15.6–26.9%)	10
d’Ambrosio et al. (2020)/multi-EU	62	39.5 (8.5)	40 (64.5)	2.0 (Mdn)	8.2 (6.3)	6; BRB-N (PASAT, SDMT, 10/36 SpaRT, SRT, WLG), WCST	<2 SDs, 2 tests	Norms	37.1% (26.1–49.7%)	12
Damasceno et al. (2020)/Brazil	42	30.5 (6.6)	32 (76.2)	2.3 (Mdn)	6.4 (4.9)	5; BRB-N (PASAT, SDMT, 10/36 SpaRT, SRT, WLG)	<1.5 SDs, 2 domains	Norms	31.0% (18.9–46.3%)	12
Deloire et al. (2005)/France	58	37.3 (9.2)	44 (75.9)	2.0 (Mdn)	2.0 (2.2)	10; BRB-N (PASAT, SDMT, 10/36 SpaRT, SRT, WLG), BNT, GNG, RFF, Stroop, WAIS-R (Similarities)	<1.67 SDs, 2 tests	HCs (*n* = 44)	44.8% (32.6–57.7%)	12
Dusankova et al. (2012)/Czech Republic	250	NR for RRMS	NR for RRMS	NR for RRMS	NR for RRMS	3; BICAMS (BVMT-R, CVLT-II, SDMT)	<1.5 SDs, 2 tests	Norms	42.4% (36.4–48.6%)	12
Eshaghi et al. (2012)/Iran	127	NR for RRMS	NR for RRMS	NR for RRMS	NR for RRMS	7; MACFIMS (BVMT-R, COWAT, CVLT-II, D-KEFS Sorting, JLO, PASAT, SDMT)	<1.5 SDs, 2 tests	HCs (*n* = 90)	42.5% (34.2–51.3%)	12
Gajofatto et al. (2016)/Italy	35	NR for tested sample	NR for tested sample	NR for tested sample	NR for tested sample	7; Attentive Numeric Matrices, Bisyllabic Word Span, Corsi Test, Numeric Span, SRT, Token Test, WLG	<2 SDs, 1 domain^*^	Norms	20.0% (9.8–36.4%)	12
Gois et al. (2021)/Brazil	24	46.7 (11.6)	14 (58.3)	2.3 (Mdn)	13.1 (7.5)	7; BRB-N (PASAT, SDMT, 10/36 SpaRT, SRT, WLG), BNT, Tower of London	<1.5 SDs, 1 domain^*^	Norms	20.8% (8.9–41.3%)	12
Goretti et al. (2014)/Italy	190	37.5 (9.9)	140 (73.7)	3.2 (1.6)	11.6 (8.4)	6; BRB-N (PASAT, SDMT, 10/36 SpaRT, SRT, WLG), Stroop	<1.67 SDs, 3 tests	Norms	40.0% (33.3–47.1%)	12
Hulst et al. (2012)/The Netherlands	36	NR for RRMS	NR for RRMS	NR for RRMS	10.8 (7.1)	5; LDST, LLT, Semantic Fluency, VLGT, WAIS-III Digit Span (Forward & Backward)	<2 SDs, 2 tests	HCs (*n* = 30)	25.0% (13.6–41.5%)	12
Iaffaldano et al. (2014)/Italy	49	38.1 (10.0)	34 (69.4)	2.6 (0.7)	13.4 (9.8)	6; BRB-N (PASAT, SDMT, 10/36 SpaRT, SRT, WLG), Stroop	<1.67 SDs, 3 tests	Norms	26.5% (16.1–40.5%)	12
Jakimovski et al. (2019)/USA	73	NR for RRMS	NR for RRMS	NR for RRMS	NR for RRMS	12; MACFIMS (BVMT-R, COWAT, CVLT-II, D-KEFS Sorting, PASAT, SDMT), Beery VMI, BNT, Clock Drawing, Semantic Fluency (Animals, Supermarket Items), WMS-R (Logical Memory)	<1.5 SDs, 2 domains	HCs (*n* = 56)	42.5% (31.7–54.0%)	10
Jandric et al. (2021)/United Kingdom	102	45.0 (Mdn); 18–60 (range)	69 (67.6)	NR	12.2 (Mdn)	5; BRB-N (PASAT, SDMT, 10/36 SpaRT, SRT, WLG)	<1.5 SDs, 2 tests	HCs (*n* = 27)	53.9% (44.2–63.3%)	12
Jonkman et al. (2015)/USA	42	43.7 (9.6)	31 (73.8)	1.8 (NR)	5.8 (5.2)	6; CVLT-II, Letter Fluency, DKEFS (Inhibition, Inhibition Switching), SDMT, PASAT	<1.67 SDs, 2 tests	Norms	21.4% (11.5–36.3%)	10
Lanzillo et al. (2006)/Italy	52	30 (Mdn); 18–46 (range)	33 (63.5)	2.0 (Mdn)	3.4 (Mdn)	14; Constructive Apraxia Test, Corsi Test, MMSE, PASAT, Raven Matrices, RCFT, Rey ST/LT, Story Recall, Stroop, Token Test, Verbal Fluency, Verbal Span, Weigl Test	<1.67 SDs, 4 tests	Norms	46.2% (33.2–59.7%)	10
Lozano-Soto et al. (2021)/Spain	91	48.6 (8.8)	63 (69.2)	2.5 (2.0)	10.4 (6.9)	6; Letter Fluency, PASAT, SDMT, Semantic Fluency, WMS-III (Word List Test Short/Long-Term)	<1.5 SDs, 1 test^*^	Norms	42.9% (33.1–53.2%)	11
Ma et al. (2017)/Canada	39	47.3 (6.0)	27 (69.2)	NR for RRMS	11.7 (NR)	7; MACFIMS (BVMT-R, COWAT, CVLT-II, D-KEFS Sorting, JLO, PASAT, SDMT)	<1.5 SDs, 2 tests	HCs (*n* = 19)	51.3% (36.0–66.4%)	12
Maarouf et al. (2017)/France	58	35.6 (8.7)	42 (72.4)	NR for RRMS	NR for RRMS	5; BRB-N (PASAT, SDMT, 10/36 SpaRT, SRT, WLG)	<2 SDs, 2 tests	HCs (*n* = 31)	36.2% (24.9–49.2%)	12
Mashayekhi et al. (2022)/Iran	71	31.4 (8.8)	53 (74.6)	1.3 (1.2)	5.5 (4.4)	7; MACFIMS (BVMT-R, COWAT, CVLT-II, D-KEFS Sorting, JLO, PASAT, SDMT)	<1.5 SDs, 2 tests	Norms	14.1% (7.7–24.2%)	10
Maubeuge et al. (2021)/France	43	NR for RRMS	NR for RRMS	NR for RRMS	NR for RRMS	7; MACFIMS (BVMT-R, COWAT, CVLT-II, D-KEFS Sorting, JLO, PASAT, SDMT)	<1.5 SDs, 2 domains	HCs (*n* = 276)	18.6% (9.6–33.0%)	12
Meijer et al. (2017)/The Netherlands	243	NR for RRMS	NR for RRMS	NR for RRMS	NR for RRMS	5; BRB-N (PASAT, SDMT, 10/36 SpaRT, SRT, WLG)	<2 SDs, 2 tests	HCs (*n* = 96)	20.2% (15.6–25.7%)	12
Migliore et al. (2017)/Italy	92	41.5 (10.7)	64 (69.6)	73 ≤ 1.5; 19 2–2.5	9.5 (Mdn)	7; MACFIMS (BVMT-R, COWAT, CVLT-II, D-KEFS Sorting, JLO, PASAT, SDMT)	<1.5 SDs, 2 tests	HCs (*n* = 42)	51.1% (41.0–61.1%)	14
Moccia et al. (2016)/Italy	155	32.1 (8.5)	99 (63.9)	1.8 (0.4)	3.1 (2.5)	5; BRB-N (PASAT, SDMT, 10/36 SpaRT, SRT, WLG)	<2 SDs, 3 tests	Norms	26.5% (20.1–33.9%)	11
Niccolai et al. (2015)/Italy	192	41.4 (10.8)	142 (74.0)	2.7 (1.7)	12.7 (8.9)	5; BRB-N (PASAT, SDMT, 10/36 SpaRT, SRT, WLG)	<1.67 SDs, 2 tests	Norms	24.0% (18.4–30.5%)	12
Ozkul et al. (2020)/Turkey	96	NR for RRMS	NR for RRMS	NR for RRMS	NR for RRMS	5; BRB-N (PASAT, SDMT, 10/36 SpaRT, SRT, WLG)	<2 SDs, 2 tests	Norms	47.9% (38.1–57.9%)	12
Patti et al. (2009)/Italy	550	33.4 (8.3)	362 (65.8)	1.8 (1.0)	5.0 (5.3)	6; BRB-N (PASAT, SDMT, 10/36 SpaRT, SRT, WLG), Stroop	<1.67 SDs, 3 tests	Norms	22.0% (18.7–25.7%)	13
Portaccio et al. (2009a)/Italy	85	43.0 (8.4)	58 (68.2)	1.7 (1.0)	15.8 (9.6)	6; BRB-N (PASAT, SDMT, 10/36 SpaRT, SRT, WLG), Stroop	<2 SDs, 2 tests	Norms	32.9% (23.8–43.6%)	12
Portaccio et al. (2009b)/Italy	116	43.1 (9.1)	81 (69.8)	1.7 (1.2)	15.9 (9.3)	6; BRB-N (PASAT, SDMT, 10/36 SpaRT, SRT, WLG), Stroop	<1.67 SDs, 2 tests	Norms	44.8% (36.0–53.9%)	12
Preziosa et al. (2016)/multi-EU	61	39.7 (8.5)	40 (65.6)	1.5 (Mdn)	8.2 (NR)	6; BRB-N (PASAT, SDMT, 10/36 SpaRT, SRT, WLG), WCST	<2 SDs, 2 tests	Norms	37.7% (26.5–50.4%)	12
Rimkus et al. (2011)/Brazil	23	32.0 (9.2)	15 (65.2)	1.4 (1.2)	2.4 (1.4)	16; BNT, BVMT, HVLT, Logical Memory, RCFT Copy/Recall, SDMT, Stroop, TMT, Verbal Fluency, WAIS-III (Block Design, Digit Span Forward & Backward, Letter-Number Sequencing, Vocabulary), WCST	<1.67 SDs, 3 tests	Norms	47.8% (28.8–67.5%)	12
Rimkus et al. (2019)/The Netherlands	124	NR for RRMS	NR for RRMS	NR for RRMS	NR for RRMS	7; CST, MCT, SDMT, 10/36 SpaRT, SRT, Stroop, WLG	<2 SDs, 2 domains	None	11.3% (6.8–18.2%)	12
Rocca et al. (2014)/Italy	42	39.6 (8.5)	23 (54.8)	2.0 (Mdn)	7.7 (NR)	6; BRB-N (PASAT, SDMT, 10/36 SpaRT, SRT, WLG), WCST	<1.67 SDs, 2 tests	Norms	47.6% (33.2–62.5%)	12
Ruano et al. (2017)/Italy	759	39.9 (10.2)	529 (69.7)	2.0 (Mdn)	11.2 (8.4)	6; BRB-N (PASAT, SDMT, 10/36 SpaRT, SRT, WLG), Stroop	<1.67 SDs, 2 domains	Norms	44.5% (41.0–48.1%)	13
Sacco et al. (2015)/Italy	46	39.6 (7.7)	29 (63.0)	2.5 (Mdn)	11.7 (6.6)	6; BRB-N (PASAT, SDMT, 10/36 SpaRT, SRT, WLG), Stroop	<2 SDs, 2 tests	Norms	43.5% (30.0–57.9%)	12
Schoonhoven et al. (2019)/The Netherlands	59	NR for RRMS	NR for RRMS	NR for RRMS	NR for RRMS	8; BRB-N (PASAT, SDMT, 10/36 SpaRT, SRT, WLG), CST, MST, Stroop	<2 SDs, 2 domains	HCs (*n* = 34)	28.8% (18.7–41.6%)	11
Skorve et al. (2023)/Norway	49	38.7 (10.7)	34 (69.4)	1.3 (0.9)	2.1 (1.3)	3; BICAMS (BVMT-R, CVLT-II, SDMT)	<1.5 SDs, 1 test^*^	HCs (*n* = 68)	10.2% (4.3–22.3%)	10
Talebi et al. (2022)/Iran	91	31.6 (8.6)	65 (71.4)	1.3 (1.3)	5.9 (4.6)	7; MACFIMS (BVMT-R, COWAT, CVLT-II, D-KEFS Sorting, JLO, PASAT, SDMT)	<1.5 SDs, 2 tests	Norms	19.8% (12.8–29.2%)	12
Topcular et al. (2012)/Turkey	51	37.9 (9.8)	40 (78.4)	3.3 (1.5)	8.6 (6.7)	5; BRB-N (PASAT, SDMT, 10/36 SpaRT, SRT, WLG)	<1.67 SDs, 2 tests	Norms	41.2% (28.6–55.0%)	12
Van Schependom et al. (2014)/Belgium	144	44.2 (11.1)	95 (66.0)	2.8 (2.4)	10.5 (8.8)	4; NSBMS (COWAT, PASAT, 7/24 SpaRT, SRT)	<1.67 SDs, 2 tests	Norms	22.9% (16.8–30.5%)	11
Winter et al. (2021)/United Kingdom	40	58.1 (8.1)	27 (67.5)	2.9 (2.9)	27.0 (8.0)	12; BIRT Memory and Information Processing Battery (Design Learning, Figure Recall, List Learning, Speed of Information Processing, Story Recall), DKEFS (Inhibition, Inhibition Switching, Verbal Fluency), PASAT, WAIS-IV (Coding, Letter-Number Sequencing), Semantic Fluency	<1.67 SDs, 2 tests	Norms	60.0% (44.3–73.8%)	12
Zhang et al. (2016)/China	39	38.3 (9.1)	23 (59.0)	2.2 (1.6)	7.7 (6.0)	7; AVLT-Chinese, BVMT-R, JLO, PASAT, SDMT, TMT, Verbal Fluency	<1.5 SDs, 2 domains	HCs (*n* = 29)	35.9% (22.5–51.9%)	12

*AAT* Aachen Aphasia Test, *AVLT-Chinese* Auditory Verbal Learning Test–Chinese, *Beery VMI* Beery-Buktenica Developmental Test of Visual-Motor Integration, *BICAMS* Brief International Cognitive Assessment for Multiple Sclerosis, *BIRT* Brain Injury and Rehabilitation Trust, *BNT* Boston Naming Test, *BRB-N* Brief Repeatable Battery of Neuropsychological Tests, *BVMT-R* Brief Visuospatial Memory Test–Revised, *CIm* cognitive impairment, *CI* confidence interval, *COWAT* Controlled Oral Word Association Test, *CST* Concept Shifting Test, *CVLT-II* California Verbal Learning Test–Second Edition, *DD* disease duration, *D-KEFS* Delis-Kaplan Executive Function System, *EDSS* Expanded Disability Status Scale, *GNG* Go-No-Go, *HCs* healthy controls, *HVLT* Hopkins Verbal Learning Test, *JLO* Judgement of Line Orientation, *MACFIMS* Minimal Assessment of Cognitive Function in Multiple Sclerosis, *MCT* Memory Comparison Test, *MMSE* Mini-Mental State Examination, *NR* not reported, *NSBMS* Neuropsychological Screening Battery for Multiple Sclerosis, *PASAT* Paced Auditory Serial Addition Test, *RCFT* Rey Complex Figure Test, *Rey LT/ST* Rey Long/Short-Term Test, *RFF* Ruff Figural Fluency Test, *RRMS* relapsing-remitting multiple sclerosis, *SDs* standard deviations, *SDMT* Symbol Digit Modalities Test, *SpaRT* Spatial Recall Test, *SRT* Selective Reminding Test, *TMT* Trail Making Test, *VLGT* Verbale Leer en Geheugen Taak, *WAIS-III* Wechsler Adult Intelligence Test–Third Edition, *WAIS-IV* Wechsler Adult Intelligence Test–Fourth Edition, *WAIS-R* Wechsler Adult Intelligence Test–Revised, *WCST* Wisconsin Card Sorting Test, *WLG* Word List Generation, *WMS-III* Wechsler Memory Scale–Third Edition, *WMS-R* Wechsler Memory Scale–Revised, *WTET* Weight and Time Estimation Test

^*^Definition of impairment only required one reduced test but prevalence rates for two impaired tests are reported by authors and presented in the above table. *N* refers to the number of participants with RRMS only. The 95% confidence intervals for the cognitive impairment prevalence rates were derived from the random-effect meta-analysis. Study quality was assessed using the modified QUADAS tool

Demographic and clinical characteristics were variably reported using means or medians, and some studies which recruited mixed samples did not report summary statistics for the subset of participants with RRMS. Thirty-eight (76%) studies reported sex of RRMS participants, with 69% of these being female overall. Mean age of RRMS participants was reported or calculable in 36 (72%) studies and ranged from 30.5 to 58.1 years. Mean disease duration of RRMS participants was reported or calculable in 33 (66%) studies and ranged from 2.1 to 27.0 years. Of studies which used the Expanded Disability Status Scale (EDSS) to measure neurological disability, means of RRMS participants were reported or calculable in 26 (52%) studies and ranged from 1.3 to 4.2, and medians were reported in 11 (22%) studies and ranged from 1.5 to 3.5.

Many studies used standardized batteries developed for use in MS populations. The most common was the Brief Repeatable Battery of Neuropsychological Tests (BRB-N; Bever et al., 1995) used in 29 (58%) studies, which served as a standalone battery for determining CIm in 12 of these. Seven (14%) studies used the Minimal Assessment of Cognitive Function in MS (MACFIMS; Benedict et al., 2002), three (6%) used the Brief International Cognitive Assessment for MS (BICAMS; Benedict et al., 2012), and two (4%) used the Neuropsychological Screening Battery for MS (NSBMS; Bobholz & Rao, 2003). Forty-eight studies (96%) included a verbal fluency measure, though the type (e.g., phonemic or semantic fluency) differed across studies. Beyond this, the most frequently used test was the Symbol Digit Modalities Test (SDMT) used in 44 (88%) studies, followed by the Paced Auditory Serial Addition Test (PASAT) used in 43 (86%) studies. The number of tests administered ranged from 3 to 16. Most studies administered five (*k* = 13; 26%), six (*k* = 16; 32%), or seven (*k* = 10; 20%) tests in total, with seven studies (14%) administering more than six tests and four studies (8%) administering fewer than five tests.

The definitions of CIm varied. Selected studies used one of three cut-off points to indicate impaired performance on a test: 17 (34%) studies used a cut-off of 1.5 SDs (i.e., 7th percentile) below the comparator group’s mean, 17 (34%) used a cut-off of 1.67 SDs (i.e., 5th percentile), and 16 (32%) used a cut-off of 2 SDs (i.e., 2nd percentile). Studies differed in whether they described their criteria as dependent on the number of ‘tests’ or ‘domains’ which were impaired. In some cases, this terminology was functionally equivalent; for instance, many studies used standardized batteries which allocate the same test(s) to each cognitive domain. Most studies (*n* = 38; 76%) required impairment in at least two tests or cognitive domains for a participant to be considered impaired, while six (12%) required three tests, and one (2%) required four. Five (10%) studies only required one impaired test for CIm but reported the number of participants with two or more impaired tests; this data was extracted for the analyses. Thirty-five (70%) studies compared RRMS participants’ performances against published normative values and the remaining 15 (30%) recruited healthy controls.

### Study Quality

Study quality was generally low when examined according to criteria developed for prevalence studies (Table 2 of the Supplement). Only one study (2%) achieved the minimum score for acceptable quality on the modified QUADAS (i.e., 14/19; Broen et al., 2016). There was little variation in scores, with most studies (*k* = 37; 74%) earning 12 points generally across the same criteria. Several details regarding the representativeness of the sample were rarely reported: no studies used random sampling, only one study (2%) made a statement to indicate the representativeness of their sample, one study (2%) described recruitment non-responders or reasons for non-responding, and three studies (6%) reported the response rate.

### CIm Prevalence

**Fig. 2:**
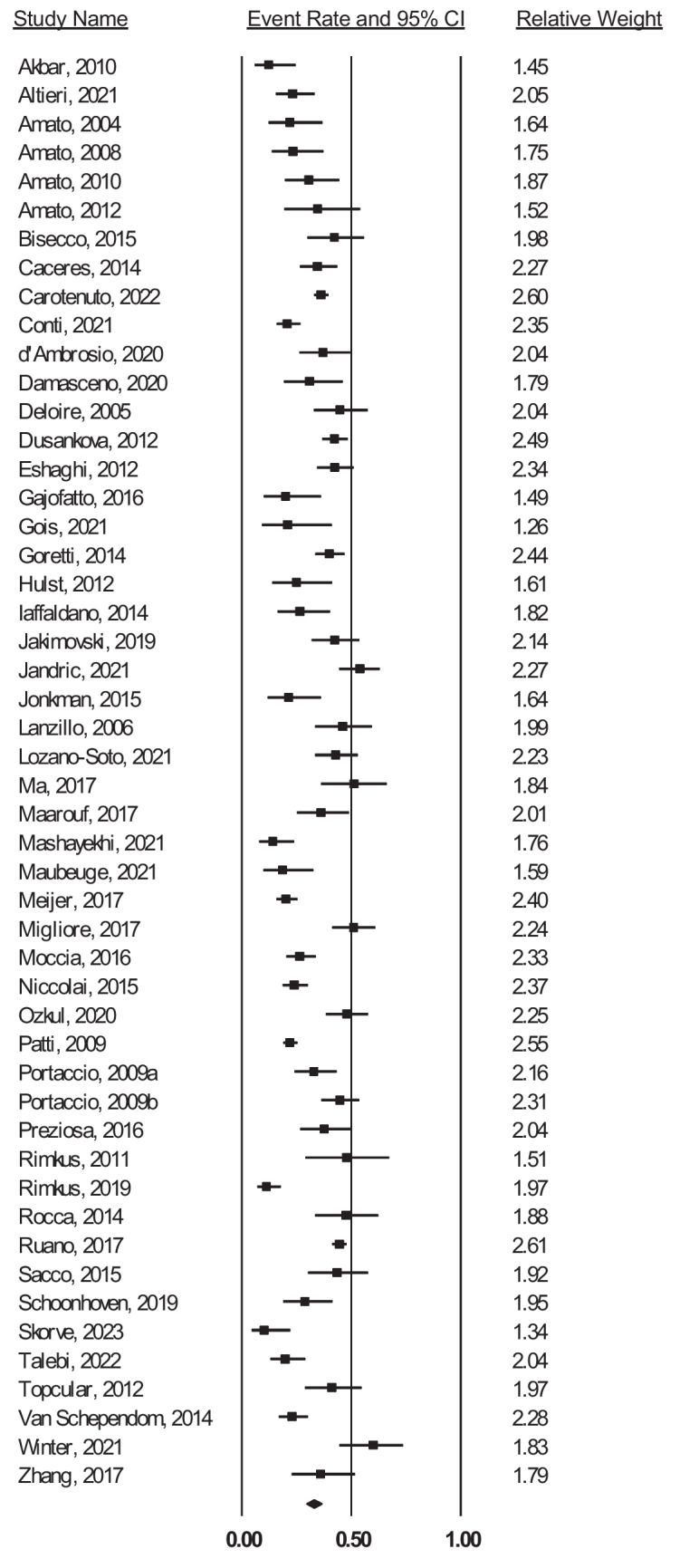
Forest plot of prevalence rates and weights of selected studies

The pooled prevalence of CIm (defined as evidence of relatively reduced performance across at least two cognitive domains) was 32.5% (95% CI 29.3–36.0%) across 5859 people with RRMS. There was substantial heterogeneity between studies (*Q*_49_ = 309.62, *p* < .001, *I*^2^ = 84.17, τ^2^ = 0.23), with reported prevalence rates ranging from 10.2 to 60.0%. The forest plot depicting prevalence and 95% CIs is displayed in Fig. 2. The funnel plot (Fig. 3 of the Supplement) was generally symmetric, Egger’s test was non-significant (*p* = .104), and the trim-and-fill procedure did not suggest trimming any studies; thus, there was no evidence of publication bias.

### Moderators of CIm Prevalence

Results of moderator analyses are summarized in Table 2. Meta-regression analyses indicated that older age (*k* = 36, *b* = 0.034, *Q*_1_ = 5.66, *p* = .017) and greater disease duration (*k* = 33, *b* = 0.042, *Q*_1_ = 7.96, *p* = .005) were significantly associated with higher prevalence of CIm. Studies which administered more tests also reported significantly greater CIm prevalence (*k* = 50, *b* = 0.073, *Q*_1_ = 5.10, *p* = .024). There were no significant effects of year of publication (*k* = 50, *b* = −0.013, *Q*_1_ = 0.62, *p* = .431), RRMS sample size (*k* = 50, *b* = 0.000, *Q*_1_ = 0.02, *p* = .875), or the proportion of female participants (*k* = 38, *b* = −0.018, *Q*_1_ = 1.15, *p* = .283).

**Table 2:** Results of random effect meta-analysis and moderator analyses

	*k*	Prevalence (95% CI)	*I*^2^	τ^2^	*Q*	*p*
Overall	50	32.5 (29.3–36.0)	84.17	0.23	309.62	<.001
EDSS	38				3.39	.066
<2.0	16	29.6 (23.7–36.2)	83.49	0.29	90.87	<.001
≥2.0	22	36.7 (33.0–40.7)	72.08	0.10	75.22	<.001
Cut-off for test impairment	50				2.14	.343
1.5 SDs	17	33.2 (27.7–39.1)	83.70	0.22	98.14	<.001
1.67 SDs	17	35.2 (29.2–41.7)	87.67	0.26	129.73	<.001
2 SDs	16	29.2 (24.1–34.9)	75.80	0.21	61.99	<.001
Number of tests for CIm	50				0.04	.845
2	43	32.7 (29.2–36.4)	83.39	0.22	252.89	<.001
>2	7	31.8 (24.1–40.6)	83.29	0.20	35.91	<.001
Comparison group	50				0.64	.425
Normative values	35	31.6 (27.8–35.6)	85.27	0.22	230.77	<.001
Healthy controls	15	34.8 (28.2–41.9)	81.94	0.28	77.52	<.001
Study quality	50				2.87	.090
<12	10	26.9 (20.6–34.4)	80.56	0.25	46.29	<.001
≥12	40	34.0 (30.4–37.9)	83.97	0.21	243.31	<.001

	*k*	*b* (95% CI)			*Q*	*p*
Age	36	0.034 (0.014–0.006)			5.66	.017*
Disease duration	33	0.042 (0.013–0.070)			7.96	.005*
Proportion female	38	−0.018 (−0.051–0.015)			1.15	.283
Number of tests administered	50	0.073 (0.010–0.137)			5.10	.024*
Year of publication	50	−0.013 (−0.046–0.020)			0.62	.431
Sample size	50	0.000 (−0.001–0.001)			0.02	.875

*CIm* cognitive impairment, *EDSS* Expanded Disability Status Scale, *SDs* standard deviations

*Moderator significant at *p* < .05

Though there appeared to be a trend toward greater CIm prevalence in studies with a mean or median EDSS of 2.0 or above (36.7%; 95% CI 33.0–40.7%) compared with those with EDSS below 2.0 (29.6%; 95% CI 23.7–36.2%), subgroup analyses indicated that this was non-significant (*k* = 38, *Q*_1_ = 3.39, *p* = .066). There were no significant effects of the cut-off used to indicate impaired test performance (*k* = 50, *Q*_2_ = 2.14, *p* = .343), number of impaired tests to indicate CIm (*k* = 50, *Q*_1_ = 0.04, *p* = .845), the comparison group used (*k* = 50, *Q*_1_ = 0.64, *p* = .425), or study quality (*k* = 50, *Q*_1_ = 2.87, *p* = .090).

## Discussion

The present meta-analysis estimated that approximately one third (32.5%) of adults with RRMS experience CIm. While previous meta-analytic findings have provided indication of the magnitude of CIm in RRMS (Prakash et al., 2008), our findings extend the literature by providing a quantitative estimate of the proportion of people with RRMS who are affected. Though lower than the 40–65% estimate reported by Amato and colleagues in 2006 (Amato et al., 2006), our estimate is an empirical analysis focused on RRMS which incorporated comprehensive data across 50 studies (*n* = 5859) and only included studies which adopted a stringent definition of CIm (Hancock et al., 2022).

There was substantial between-study heterogeneity, which was partially accounted for by clinical and demographic factors. Disease duration and age were associated with greater CIm prevalence, such that samples with longer disease durations and higher ages had greater CIm prevalence. These findings are consistent with a past meta-analysis (*k* = 57; *n* = 3891) by Prakash et al. (2008), which found that the magnitude of cognitive deficits (defined as the difference in cognitive performance relative to healthy controls) among people with RRMS was moderated by higher age, while greater disease duration moderated the magnitude of deficits in the domain of memory and learning only. The impact on disease duration and age on CIm prevalence is also consistent with expectations given that overall disability in RRMS tends to worsen over time even with treatment (Cree et al., 2016).

One challenge in collating CIm prevalence data across the literature are the disparate definitions of CIm used across studies. Although it would have been ideal to adopt one unified definition of CIm for the current study, this was not possible as prevalence rates for different definitions of CIm were rarely extractable from studies. We attempted to address this variability by including aspects of CIm definitions as moderators in our analyses. However, we found that differences in the definitions of CIm used across studies did not moderate prevalence rates. Specifically, prevalence rates did not vary significantly between studies which utilized cut-offs of 1.5, 1.67, or 2 SDs to indicate impaired test performance, or between those which required at least two impaired tests or at least three impaired tests for a participant to meet criteria for CIm. This may be due to our stringent and best-practice CIm definition (i.e., evidence of reduction in at least two cognitive domains) and exclusion of studies that only required impairment in one domain. Additionally, the prevalence rates reported in studies which compared RRMS participants against healthy controls (34.8%; 95% CI 28.2–41.9%) were not significantly different from those which used published normative values (31.6%; 95% CI 27.8–35.6%), supporting the use of published normative values as a valid comparison group for research purposes.

We also found that studies that used more extensive test batteries reported significantly higher CIm prevalence rates. This finding is somewhat unsurprising considering that the administration of more tests provides greater opportunity for a participant to be detected as having CIm. This is especially the case due to the definitions of CIm adopted in some research, for instance, if multiple tests are used to measure a single cognitive domain and reduction on any of these tests is considered sufficient to indicate impairment in that domain. We note that the majority of included studies administered tests relevant to cognitive changes in MS, such as the SDMT or PASAT, and many studies administered brief batteries developed specifically for people with MS (e.g., the BRB-N used in 58% of studies). While these tests and batteries have been validated to be sensitive to cognitive impairment in MS (Drake et al., 2010; Sumowski et al., 2018; Strober et al., 2009), the finding that a more comprehensive test battery is more likely detect cognitive deficits supports this as standard practice for neuropsychological assessment in clinical settings.

It was not possible to formally estimate domain-specific impairment rates across all participants as many studies did not report how many participants were impaired on each test or domain. Of the studies which did report the most frequently impaired domain, attention and information processing speed, typically measured by the SDMT and/or PASAT, was by far the most common (Altieri et al., 2021; Amato et al., 2008; Bisecco et al., 2015; Conti et al., 2021; d’Ambrosia et al., 2020; Deloire et al., 2005; Gois et al., 2021; Goretti et al, 2014; Jakimovski et al., 2019; Lozano-Soto, 2021; Mashayekhi et al., 2022; Portaccio et al., 2009a, 2009b; Preziosa et al., 2016; Rimkus et al., 2011; Rocca et al., 2014; Ruano et al., 2017; Sacco et al., 2015; Talebi et al., 2022), followed by verbal memory (Conti et al., 2021; Gajofatto et al., 2016; Goretti et al., 2014; Maubeuge et al., 2021; Skorve et al., 2023), and visual memory (Amato et al., 2004; Damasceno et al., 2020; Hulst et al., 2012), with few studies reporting executive function (Rimkus et al., 2019) and visuospatial functioning (Lanzillo et al., 2006) as most affected. This is consistent with other work indicating that attention and processing speed deficits are the most prevalent cognitive difficulty in MS (McNicholas et al., 2018).

There are several caveats to the representativeness of the estimate we obtained. Though we did not place restrictions on the recruitment setting when selecting studies, all included studies which reported this recruited from a clinical setting, and no studies based purely on community samples met inclusion criteria. Thus, our estimate is constrained to people with RRMS who attend clinical services and may not generalize to people with RRMS more generally. In light of this, the current meta-analysis may overestimate the prevalence of CIm, as people with concerns about their cognitive functioning may be more likely to present to a clinical setting, leading to selection bias (Abdelnour et al., 2017; Farias et al., 2009). Future research conducting formal neuropsychological testing in community-based samples is warranted. The scope of our study was also limited to adult-onset RRMS, and specific investigations into CIm prevalence in other MS subpopulations may be informative. Pediatric-onset MS comprises approximately 5% of all MS cases (Harding et al., 2013) and the risk of CIm may be greater than those with adult-onset MS, particularly on measures sensitive to processing speed deficits such as the SDMT and PASAT (McKay et al., 2019; Ruano et al., 2018). Given evidence of greater magnitude of CIm in the progressive disease courses (Johnen et al., 2017; Planche et al., 2016), CIm prevalence among these groups is also likely to differ from that in RRMS. Furthermore, subjective CIm may be an important aspect of patient experience distinct from objective CIm (Hughes et al., 2019; Julian et al., 2007; Kinsinger et al., 2010; Mortensen et al., 2020), which requires future examination.

Our ability to perform meta-regression analyses was also affected by the restricted ranges of obtained scores for some variables, specifically EDSS and study quality. Additionally, the study quality tool used was limited by the heterogeneity of its criteria and could only be used to create a statistical model-free count score rather than a model-based scale (Scherer & Emslander, 2024). We were thus required to generate somewhat crude binary classifications to make these variables suitable for subgroup analyses. Neither study quality nor EDSS were significant moderators in the present study. Future work which estimates CIm prevalence could improve in quality by inclusion of information regarding the possibility of recruitment bias, such as the number of people who were approached but did not participate in the study and reasons for non-participation. Potential relationships between neurological disability and CIm may also be more effectively captured by reporting separate EDSS scores for subgroups of participants with impaired or preserved cognition, allowing for between-group comparisons to be extracted meta-analytically. Otherwise, future meta-analytic work in this area may be done using an individual participant data (IPD) approach, where original participant data is collected rather than aggregate data (Tierney et al., 2023). This approach allows for more rigorous examination of moderators and has been considered a ‘gold standard’ of systematic review.

It was notable that none of the selected studies included an estimate of premorbid function as their reference point for impaired cognition. Comparing participants’ test scores to those of other groups without consideration of decline for the individual may impact the CIm prevalence rates reported (Douglas et al., 2018; Tran et al., 2021). For instance, cognitively high-functioning individuals who experience a clinically significant decline from their premorbid abilities, but whose performances on testing do not fall below the chosen normative cut-off, will fail to be classified as having CIm (Sumowski et al., 2018). Conversely, those with a low level of premorbid cognitive functioning may experience relatively mild levels of decline or even no decline, but perform below the cut-off range on testing are deemed cognitively impaired. Use of premorbid estimates may improve the accuracy of prevalence estimates in future studies.

This work has several implications. Our finding that a third of adults with RRMS have CIm is lower than previous ranges cited in the literature for people with MS, such as the 40–65% range previously reported (Amato et al., 2006). This updated, rigorous, and narrower prevalence estimate may provide clinicians with increased confidence when communicating with patients regarding the risk of cognitive difficulties, which is often a concern for newly diagnosed people with MS who are typically of working age (Day et al., 2018; Heesen et al., 2008). Importantly, our estimate is specific to those with a relapsing-remitting disease course, who comprise the majority of MS cases, rather than previous estimates which do not distinguish between relapsing-remitting and progressive courses of MS which experience more severe CIm (Johnen et al., 2017). Our finding can also facilitate management of resource allocation. MS services can make provisions with the view that a third of RRMS patients are likely to require more intensive support for cognitive functioning, such as comprehensive assessment and access to cognitive rehabilitation services. This information may help to determine priorities for funding and staffing. Furthermore, this finding establishes a benchmark for future research into risk profiles, such as factors which predict CIm, to further initiatives into prevention and management.

## Conclusion

Ultimately, our finding that approximately one third of adults with RRMS experience CIm, performing below the 7th percentile in two or more cognitive domains, is important for clinical practice. People with RRMS who have CIm are more vulnerable to poor functional outcomes than those without CIm (Bruce et al., 2010; Clemens & Langdon, 2018; Goverover et al., 2019; Rao et al., 1991b; Yazgan et al., 2021), and thus are likely to benefit from additional supports and provision of strategies to manage their symptoms. Routine cognitive testing may be appropriate for those with RRMS to identify such individuals, particularly as patient attitudes to this are positive (Mortensen et al., 2020) and it aligns with both patients’ and clinicians’ priorities for treatment (Singer et al., 2021).

## Supplementary Information

Below is the link to the electronic supplementary material.

Supplementary file1 (DOCX 48 kb)

## Data Availability

No new data was generated in this study. Relevant data appear within the article and supplementary materials. Extracted data may be shared upon reasonable request.

## References

[CR1] Abdelnour, C., Rodríguez-Gómez, O., Alegret, M., Valero, S., Moreno-Grau, S., Sanabria, Á., Hernández, I., Rosende-Roca, M., Vargas, L., Mauleón, A., Sánchez, D., Espinosa, A., Ortega, G., Pérez-Cordón, A., Diego, S., Gailhajanet, A., Guitart, M., Sotolongo-Grau, Ó., Ruiz, A., … Boada, M. (2017). Impact of recruitment methods in subjective cognitive decline. *Journal of Alzheimer’s Disease,**57*, 625–632. 10.3233/JAD-160915 28269773 10.3233/JAD-160915

[CR2] Akbar, N., Lobaugh, N. J., O’Connor, P., Moradzadeh, L., Scott, C. J., & Feinstein, A. (2010). Diffusion tensor imaging abnormalities in cognitively impaired multiple sclerosis patients. *Canadian Journal of Neurological Sciences,**37*(5), 608–614. 10.1017/S0317167100010775 10.1017/s0317167100010775 21059506

[CR3] Altieri, M., Fratino, M., Maestrini, I., Dionisi, C., Annecca, R., Vicenzini, E., & Di Piero, V. (2021). Cognitive performance in relapsing-remitting multiple sclerosis: At risk or impaired? *Dementia and Geriatric Cognitive Disorders,**49*(6), 539–543. 10.1159/000514674 10.1159/000514674 33735893

[CR4] Amato, M., Hakiki, B., Goretti, B., Rossi, F., Stromillo, M., Giorgio, A., Roscio, M., Ghezzi, A., Guidi, L., Bartolozzi, M., Portaccio, E., & De Stefano, N. (2012). Association of MRI metrics and cognitive impairment in radiologically isolated syndromes. *Neurology,**78*(5), 309–314. 10.1212/wnl.0b013e31824528c9 22262744 10.1212/WNL.0b013e31824528c9

[CR5] Amato, M., Bartolozzi, M., Zipoli, V., Portaccio, E., Mortilla, M., Guidi, L., Siracusa, G., Sorbi, S., Federico, A., & De Stefano, N. (2004). Neocortical volume decrease in relapsing-remitting MS patients with mild cognitive impairment. *Neurology,**63*(1), 89–93. 10.1212/01.wnl.0000129544.79539.d5 15249616 10.1212/01.wnl.0000129544.79539.d5

[CR6] Amato, M., Portaccio, E., Stromillo, M., Goretti, B., Zipoli, V., Siracusa, G., Battaglini, M., Giorgio, A., Bartolozzi, M., Guidi, L., Sorbi, S., Federico, A., & De Stefano, N. (2008). Cognitive assessment and quantitative magnetic resonance metrics can help to identify benign multiple sclerosis. *Neurology,**71*(9), 632–638. 10.1212/01.wnl.0000324621.58447.00 18725589 10.1212/01.wnl.0000324621.58447.00

[CR7] Amato, M. P., Portaccio, E., Goretti, B., Zipoli, V., Iudice, A., Della Pina, D., Malentacchi, G., Sabatini, S., Annunziata, P., Falcini, M., Mazzoni, M., Mortilla, M., Fonda, C., De Stefano, N., & Tu, S. S. G. (2010). Relevance of cognitive deterioration in early relapsing-remitting MS: a 3-year follow-up study. *Multiple Sclerosis,**16*(12), 1474–1482. 10.1177/1352458510380089 20729256 10.1177/1352458510380089

[CR8] Amato, M. P., Zipoli, V., & Portaccio, E. (2006). Multiple sclerosis-related cognitive changes: A review of cross-sectional and longitudinal studies. *Journal of the Neurological Sciences,**245*(1), 41–46. 10.1016/j.jns.2005.08.019 16643953 10.1016/j.jns.2005.08.019

[CR9] Benedict, R. H., Amato, M. P., Boringa, J., Brochet, B., Foley, F., Fredrikson, S., Hamalainen, P., Hartung, H., Krupp, L., Penner, I., Reder, A. T., & Langdon, D. (2012). Brief International Cognitive Assessment for MS (BICAMS): International standards for validation. *BMC Neurology, 12*, 55. https://simsrad.net.ocs.mq.edu.au/login?url=http://ovidsp.ovid.com/ovidweb.cgi?T=JS&CSC=Y&NEWS=N&PAGE=fulltext&D=med9&AN=22799620https://libkey.io/libraries/590/openurl?sid=OVID:medline&id=pmid:22799620&id=doi:10.1186%2F1471-2377-12-55&issn=1471-2377&isbn=&volume=12&issue=1&spage=55&pages=55&date=2012&title=BMC+Neurology&atitle=Brief+International+Cognitive+Assessment+for+MS+%28BICAMS%29%3A+international+standards+for+validation.&aulast=Benedict 10.1186/1471-2377-12-55 PMC3607849 22799620

[CR10] Benedict, R. H., Fischer, J. S., Archibald, C. J., Arnett, P. A., Beatty, W. W., Bobholz, J., Chelune, G. J., Fisk, J. D., Langdon, D. W., Caruso, L., Foley, F., LaRocca, N. G., Vowels, L., Weinstein, A., DeLuca, J., Rao, S. M., & Munschauer, F. (2002). Minimal neuropsychological assessment of MS patients: A consensus approach. *The Clinical Neuropsychologist,**16*(3), 381–397. 10.1076/clin.16.3.381.13859 12607150 10.1076/clin.16.3.381.13859

[CR11] Bever, C. T., Jr., Grattan, L., Panitch, H. S., & Johnson, K. P. (1995). The brief repeatable battery of neuropsychological tests for multiple sclerosis: A preliminary serial study. *Multiple Sclerosis Journal,**1*(3), 165–169. 10.1177/135245859500100306 9345448 10.1177/135245859500100306

[CR12] Bisecco, A., Rocca, M., Pagani, E., Mancini, L., Enzinger, C., Gallo, A., Vrenken, H., Stromillo, M. L., Copetti, M., Thomas, D., Fazekas, F., Tedeschi, G., Barkhof, F., De Stefano, N., & Filippi, M. (2015). Connectivity-based parcellation of the thalamus in multiple sclerosis and its implications for cognitive impairment: A multicenter study. *Human Brain Mapping, 36*(7). 10.1002/hbm.22809 10.1002/hbm.22809 PMC6869750 25873194

[CR13] Bobholz, J. A., & Rao, S. M. (2003). Cognitive dysfunction in multiple sclerosis: A review of recent developments. *Current Opinion in Neurology,**16*(3), 283–288. 10.1097/01.wco.0000073928.19076.84 12858063 10.1097/01.wco.0000073928.19076.84

[CR14] Borenstein, M., Hedges, L., Higgins, J., & Rothstein, H. (2013). *Comprehensive meta-analysis version 3*. Biostat.

[CR15] Broen, M. P., Braaksma, M. M., Patijn, J., & Weber, W. E. (2012). Prevalence of pain in Parkinson’s disease: A systematic review using the modified QUADAS tool. *Movement Disorders,**27*(4), 480–484. 10.1002/mds.24054 22231908 10.1002/mds.24054

[CR16] Broen, M. P. G., Narayen, N. E., Kuijf, M. L., Dissanayaka, N. N. W., & Leentjens, A. F. G. (2016). Prevalence of anxiety in Parkinson’s disease: A systematic review and meta-analysis. *Movement Disorders,**31*(8), 1125–1133. 10.1002/mds.26643 27125963 10.1002/mds.26643

[CR17] Bruce, J. M., Hancock, L. M., Arnett, P., & Lynch, S. (2010). Treatment adherence in multiple sclerosis: Association with emotional status, personality, and cognition. *Journal of Behavioral Medicine,**33*(3), 219–227. 10.1007/s10865-010-9247-y 20127401 10.1007/s10865-010-9247-y

[CR18] Caceres, F., Vanotti, S., Benedict, R. H., & RELACCEM Work Group. (2014). Cognitive and neuropsychiatric disorders among multiple sclerosis patients from Latin America: Results of the RELACCEM study. *Multiple Sclerosis and Related Disorders,**3*(3), 335–340. 10.1016/j.msard.2013.10.007 25876470 10.1016/j.msard.2013.10.007

[CR19] Carotenuto, A., Valsasina, P., Schoonheim, M. M., Geurts, J. J. G., Barkhof, F., Gallo, A., Tedeschi, G., Tommasin, S., Pantano, P., Filippi, M., & Rocca, M. A. (2022). Investigating functional network abnormalities and associations with disability in multiple sclerosis. *Neurology,**99*(22), e2517–e2530. 10.1212/wnl.0000000000201264 36096690 10.1212/WNL.0000000000201264

[CR20] Clemens, L., & Langdon, D. (2018). How does cognition relate to employment in multiple sclerosis? A systematic review. *Multiple Sclerosis and Related Disorders,**26*, 183–191. 10.1016/j.msard.2018.09.018 30268039 10.1016/j.msard.2018.09.018

[CR21] Conti, L., Preziosa, P., Meani, A., Vizzino, C., Riccitelli, G., Pagani, E., Valsasina, P., Marchesi, O., Filippi, M., & Rocca, M. A. (2021). Unraveling the substrates of cognitive impairment in multiple sclerosis: The contribution of a multiparametric structural and functional mri approach. *Multiple Sclerosis Journal,**26*, 513. 10.1111/ene.15023 10.1111/ene.15023 34255918

[CR22] Cree, B. A., Gourraud, P. A., Oksenberg, J. R., Bevan, C., Crabtree-Hartman, E., Gelfand, J. M., Goodin, D. S., Graves, J., Green, A. J., Mowry, E., Okuda, D. T., Pelletier, D., von Büdingen, H. C., Zamvil, S. S., Agrawal, A., Caillier, S., Ciocca, C., Gomez, R., Kanner, R., … & Hauser, S. L. (2016). Long-term evolution of multiple sclerosis disability in the treatment era. *Annals of Neurology,**80*(4), 499–510. 10.1002/ana.24747 27464262 10.1002/ana.24747 PMC5105678

[CR23] Damasceno, A., Pimentel-Silva, L. R., Damasceno, B. P., & Cendes, F. (2020). Cognitive trajectories in relapsing-remitting multiple sclerosis: A longitudinal 6-year study. *Multiple Sclerosis Journal,**26*(13), 1740–1751. 10.1177/1352458519878685 31603042 10.1177/1352458519878685

[CR24] d’Ambrosio, A., Valsasina, P., Gallo, A., De Stefano, N., Pareto, D., Barkhof, F., Ciccarelli, O., Enzinger, C., Tedeschi, G., Stromillo, M. L., Arévalo, M. J., Hulst, H. E., Muhlert, N., Koini, M., Filippi, M., & Rocca, M. A. (2020). Reduced dynamics of functional connectivity and cognitive impairment in multiple sclerosis. *Multiple Sclerosis Journal,**26*(4), 476–488. 10.1177/1352458519837707 30887862 10.1177/1352458519837707

[CR25] Day, G. S., Rae-Grant, A., Armstrong, M. J., Pringsheim, T., Cofield, S. S., & Marrie, R. A. (2018). Identifying priority outcomes that influence selection of disease-modifying therapies in MS. *Neurology Clinical Practice,**8*(3), 179–185. 10.1212/cpj.0000000000000449 30105155 10.1212/CPJ.0000000000000449 PMC6075970

[CR26] Deeks, J. J., Higgins, J. P. T., & Altman, D. G. (2019). *Analysing data and undertaking meta-analyses* (Vol. 2nd Edition). John Wiley & Sons.

[CR27] Deloire, M. S., Salort, E., Bonnet, M., Arimone, Y., Boudineau, M., Amieva, H., Barroso, B., Ouallet, J. C., Pachai, C., Galliaud, E., Petry, K. G., Dousset, V., Fabrigoule, C., & Brochet, B. (2005). Cognitive impairment as marker of diffuse brain abnormalities in early relapsing remitting multiple sclerosis. *Journal of Neurology, Neurosurgery & Psychiatry,**76*(4), 519–526. 10.1136/jnnp.2004.045872 15774439 10.1136/jnnp.2004.045872 PMC1739602

[CR28] Douglas, K. M., Gallagher, P., Robinson, L. J., Carter, J. D., McIntosh, V. V., Frampton, C. M., Watson, S., Young, A. H., Ferrier, I. N., & Porter, R. J. (2018). Prevalence of cognitive impairment in major depression and bipolar disorder. *Bipolar Disorders,**20*(3), 260–274. 10.1111/bdi.12602 29345037 10.1111/bdi.12602

[CR29] Drake, A. S., Weinstock-Guttman, B., Morrow, S. A., Hojnacki, D., Munschauer, F. E., & Benedict, R. H. (2010). Psychometrics and normative data for the Multiple Sclerosis Functional Composite: Replacing the PASAT with the Symbol Digit Modalities Test. *Multiple Sclerosis,**16*(2), 228–237. 10.1177/1352458509354552 20028710 10.1177/1352458509354552

[CR30] Dusankova, J. B., Kalincik, T., Havrdova, E., & Benedict, R. H. B. (2012). Cross cultural validation of the minimal assessment of cognitive function in multiple sclerosis (MACFIMS) and the brief international cognitive assessment for multiple sclerosis (BICAMS). *Clinical Neuropsychologist,**26*(7), 1186–1200. 10.1080/13854046.2012.725101 23034066 10.1080/13854046.2012.725101

[CR31] Duval, S., & Tweedie, R. (2000). Trim and fill: A simple funnel-plot-based method of testing and adjusting for publication bias in meta-analysis. *Biometrics,**56*(2), 455–463. http://www.jstor.org/stable/2676988 10.1111/j.0006-341x.2000.00455.x 10877304

[CR32] Eshaghi, A., Riyahi-Alam, S., Roostaei, T., Haeri, G., Aghsaei, A., Aidi, M. R., Pouretemad, H. R., Zarei, M., Farhang, S., Saeedi, R., Nazeri, A., Ganjgahi, H., Etesam, F., Azimi, A. R., Benedict, R. H. B., & Sahraian, M. A. (2012). Validity and reliability of a Persian translation of the Minimal Assessment of Cognitive Function in Multiple Sclerosis (MACFIMS). *Clinical Neuropsychologist,**26*(6), 975–984. 10.1080/13854046.2012.694912 22681459 10.1080/13854046.2012.694912

[CR33] Farias, S. T., Mungas, D., Reed, B. R., Harvey, D., & DeCarli, C. (2009). Progression of mild cognitive impairment to dementia in clinic- vs community-based cohorts. *Archives of Neurology,**66*(9), 1151–1157. 10.1001/archneurol.2009.106 19752306 10.1001/archneurol.2009.106 PMC2863139

[CR34] Gajofatto, A., Turatti, M., Bianchi, M., Forlivesi, S., Gobbin, F., Azzara, A., Monaco, S., & Benedetti, M. (2016). Benign multiple sclerosis: Physical and cognitive impairment follow distinct evolutions. *Acta Neurologica Scandinavica,**133*(3), 183–191. 10.1111/ane.12442 26009804 10.1111/ane.12442

[CR35] Gois, L. C. P., Pimentel-Silva, L. R., Damasceno, B. P., & Damasceno, A. (2021). Associations between cognitive and clinical disability across MS subtypes: The role of the underlying brain damage. *Multiple Sclerosis and Related Disorders,**48*, 102701. 10.1016/j.msard.2020.102701 33477004 10.1016/j.msard.2020.102701

[CR36] Goretti, B., Viterbo, R., Portaccio, E., Niccolai, C., Hakiki, B., Piscolla, E., Iaffaldano, P., Trojano, M., & Amato, M. (2014). Anxiety state affects information processing speed in patients with multiple sclerosis. *Neurological Sciences,**35*(4), 559–563. 10.1007/s10072-013-1544-0 24072658 10.1007/s10072-013-1544-0

[CR37] Goverover, Y., Chiaravalloti, N., & DeLuca, J. (2019). Money management in multiple sclerosis: The role of cognitive, motor, and affective factors [Original Research]. *Frontiers in Neurology, 10*(1128). 10.3389/fneur.2019.01128 10.3389/fneur.2019.01128 PMC6819515 31708860

[CR38] Hancock, L. M., Hermann, B., Schoonheim, M. M., Hetzel, S. J., Brochet, B., & DeLuca, J. (2022). Comparing diagnostic criteria for the diagnosis of neurocognitive disorders in multiple sclerosis. *Multiple Sclerosis and Related Disorders,**58*, 103479. 10.1016/j.msard.2021.103479 35033839 10.1016/j.msard.2021.103479

[CR39] Harding, K. E., Liang, K., Cossburn, M. D., Ingram, G., Hirst, C. L., Pickersgill, T. P., Te Water Naude, J., Wardle, M., Ben-Shlomo, Y., & Robertson, N. P. (2013). Long-term outcome of paediatric-onset multiple sclerosis: A population-based study. *Journal of Neurology, Neurosurgery & Psychiatry,**84*(2), 141. 10.1136/jnnp-2012-303996 23154123 10.1136/jnnp-2012-303996

[CR40] Heesen, C., Böhm, J., Reich, C., Kasper, J., Goebel, M., & Gold, S. M. (2008). Patient perception of bodily functions in multiple sclerosis: Gait and visual function are the most valuable. *Multiple Sclerosis Journal,**14*(7), 988–991. 10.1177/1352458508088916 18505775 10.1177/1352458508088916

[CR41] Hughes, A. J., Bhattarai, J., Paul, S., & Beier, M. (2019). 2019/05/01/). Depressive symptoms and fatigue as predictors of objective-subjective discrepancies in cognitive function in multiple sclerosis. *Multiple Sclerosis and Related Disorders,**30*, 192–197. 10.1016/j.msard.2019.01.055 30797133 10.1016/j.msard.2019.01.055 PMC7282884

[CR42] Hulst, H. E., Schoonheim, M. M., Roosendaal, S. D., Popescu, V., Schweren, L. J., van der Werf, Y. D., Visser, L. H., Polman, C. H., Barkhof, F., & Geurts, J. J. (2012). Functional adaptive changes within the hippocampal memory system of patients with multiple sclerosis. *Hum Brain Mapp,**33*(10), 2268–2280. 10.1002/hbm.21359 21898674 10.1002/hbm.21359 PMC6869948

[CR43] Iaffaldano, P., Viterbo, R. G., Goretti, B., Portaccio, E., Amato, M. P., & Trojano, M. (2014). Emotional and neutral verbal memory impairment in multiple sclerosis. *Multiple Sclerosis,**1*, 408–409. 10.1016/j.jns.2014.03.038 10.1016/j.jns.2014.03.038 24713509

[CR44] Jakimovski, D., Weinstock-Guttman, B., Roy, S., Jaworski, M., Hancock, L., Nizinski, A., Srinivasan, P., Fuchs, T. A., Szigeti, K., Zivadinov, R., & Benedict, R. H. (2019). Cognitive profiles of aging in multiple sclerosis. *Frontiers in Aging Neuroscience*. 10.3389/fnagi.2019.00105 31133845 10.3389/fnagi.2019.00105 PMC6524468

[CR45] Jandric, D., Lipp, I., Paling, D., Rog, D., Castellazzi, G., Haroon, H., Parkes, L., Parker, G., Tomassini, V., & Muhlert, N. (2021). Mechanisms of network changes in cognitive impairment in multiple sclerosis. *Neurology,**97*, 19. 10.1212/WNL.0000000000012834 10.1212/WNL.0000000000012834 PMC8601205 34649879

[CR46] Johnen, A., Landmeyer, N. C., Bürkner, P.-C., Wiendl, H., Meuth, S. G., & Holling, H. (2017). Distinct cognitive impairments in different disease courses of multiple sclerosis—A systematic review and meta-analysis. *Neuroscience & Biobehavioral Reviews,**83*, 568–578. 10.1016/j.neubiorev.2017.09.005 28890199 10.1016/j.neubiorev.2017.09.005

[CR47] Jonkman, L., Rosenthal, D. M., Sormani, M. P., Miles, L., Herbert, J., Grossman, R. I., & Inglese, M. (2015). Gray matter correlates of cognitive performance differ between relapsing-remitting and primary-progressive multiple sclerosis. *Multiple Sclerosis,**1*, 362. 10.1371/journal.pone.0129380 10.1371/journal.pone.0129380 PMC4616346 26485710

[CR48] Julian, L., Merluzzi, N. M., & Mohr, D. C. (2007). The relationship among depression, subjective cognitive impairment, and neuropsychological performance in multiple sclerosis. *Multiple Sclerosis Journal,**13*(1), 81–86. 10.1177/1352458506070255 17294615 10.1177/1352458506070255

[CR49] Kinsinger, S. W., Lattie, E., & Mohr, D. C. (2010). Relationship between depression, fatigue, subjective cognitive impairment, and objective neuropsychological functioning in patients with multiple sclerosis. *Neuropsychology,**24*(5), 573–580. 10.1037/a0019222 20804245 10.1037/a0019222 PMC2933087

[CR50] Kurtzke, J. F. (1983). Rating neurologic impairment in multiple sclerosis: An Expanded Disability Status Scale (EDSS). *Neurology,**33*(11), 1444–1452. 10.1212/wnl.33.11.1444 6685237 10.1212/wnl.33.11.1444

[CR51] Lanzillo, R., Prinster, A., Scarano, V., Liuzzi, R., Coppola, G., Florio, C., Salvatore, E., Schiavone, V., Brunetti, A., Muto, M., Orefice, G., Alfano, B., Bonavita, V., & Morra, V. B. (2006). Neuropsychological assessment, quantitative MRI and ApoE gene polymorphisms in a series of MS patients treated with IFN beta-1b. *Journal of the Neurological Sciences,**245*(1–2), 141–145. 10.1016/j.jns.2005.08.023 16626758 10.1016/j.jns.2005.08.023

[CR52] Leboeuf-Yde, C., & Lauritsen, J. M. (1995). The prevalence of low back pain in the literature. A structured review of 26 Nordic studies from 1954 to 1993. *Spine (Phila Pa 1976),**20*(19), 2112–2118. 10.1097/00007632-199510000-00009 8588168 10.1097/00007632-199510000-00009

[CR53] Leray, E., Moreau, T., Fromont, A., & Edan, G. (2016). Epidemiology of multiple sclerosis. *Revue Neurologique,**172*(1), 3–13. 10.1016/j.neurol.2015.10.006 26718593 10.1016/j.neurol.2015.10.006

[CR54] Lozano-Soto, E., Cruz-López, Á., Gutiérrez, R., González, M., Sanmartino, F., Rashid-Lopez, R., Espinosa-Rosso, R., Forero, L., & González-Rosa, J. J. (2021). Predicting neuropsychological impairment in relapsing remitting multiple sclerosis: The Role of clinical measures, treatment, and neuropsychiatry symptoms. *Archives of clinical neuropsychology : the official journal of the National Academy of Neuropsychologists,**36*(4), 475–484. 10.1093/arclin/acaa088 33067616 10.1093/arclin/acaa088

[CR55] Ma, A. Y., Vitorino, R. C., Hojjat, S. P., Mulholland, A. D., Zhang, L., Lee, L., Carroll, T. J., Cantrell, C. G., Figley, C. R., & Aviv, R. I. (2017). The relationship between white matter fiber damage and gray matter perfusion in largescale functionally defined networks in multiple sclerosis. *Multiple Sclerosis Journal,**23*, 1884–1892. 10.1177/1352458517691149 28178867 10.1177/1352458517691149

[CR56] Maarouf, A., Audoin, B., Pariollaud, F., Gherib, S., Rico, A., Soulier, E., Confort-Gouny, S., Guye, M., Schad, L., Pelletier, J., Ranjeva, J.-P., & Zaaraoui, W. (2017). Increased total sodium concentration in gray matter better explains cognition than atrophy in MS. *Neurology,**88*(3), 289–295. 10.1212/WNL.0000000000003511 27974643 10.1212/WNL.0000000000003511

[CR57] Mashayekhi, F., Sadigh-Eteghad, S., Naseri, A., Asadi, M., Abbasi Garravnd, N., & Talebi, M. (2022). ApoE4-positive multiple sclerosis patients are more likely to have cognitive impairment: A cross-sectional study. *Neurol Sci,**43*(2), 1189–1196. 10.1007/s10072-021-05383-z 34120271 10.1007/s10072-021-05383-z

[CR58] Maubeuge, N., Deloire, M. S. A., Brochet, B., Ehrle, N., Charre-Morin, J., Saubusse, A., Ruet, A., BICAFMS Study Investigators. (2021). Validation of the French version of the Minimal Assessment of Cognitive Function in Multiple Sclerosis (MACFIMS). *Multiple Sclerosis and Related Disorders,**48*, 102692. 10.1016/j.msard.2020.102692 33352358 10.1016/j.msard.2020.102692

[CR59] McIntosh-Michaelis, S. A., Roberts, M. H., Wilkinson, S. M., Diamond, I. D., McLellan, D. L., Martin, J. P., & Spackman, A. J. (1991). The prevalence of cognitive impairment in a community survey of multiple sclerosis. *British Journal of Clinical Psychology,**30*(4), 333–348. 10.1111/j.2044-8260.1991.tb00954.x 1777755 10.1111/j.2044-8260.1991.tb00954.x

[CR60] McKay, K. A., Manouchehrinia, A., Berrigan, L., Fisk, J. D., Olsson, T., & Hillert, J. (2019). Long-term cognitive outcomes in patients with pediatric-onset vs adult-onset multiple sclerosis. *JAMA Neurology,**76*(9), 1028–1034. 10.1001/jamaneurol.2019.1546 31206130 10.1001/jamaneurol.2019.1546 PMC6580443

[CR61] McNicholas, N., O’connell, K., Yap, S. M., Killeen, R. P., Hutchinson, M., & McGuigan, C. (2018). Cognitive dysfunction in early multiple sclerosis: A review. *QJM: An International Journal of Medicine,**111*(6), 359–364. 10.1093/qjmed/hcx070 28371862 10.1093/qjmed/hcx070

[CR62] Meijer, K. A., Eijlers, A. J. C., Douw, L., Uitdehaag, B. M., Barkhof, F., Geurts, J. J., & Schoonheim, M. M. (2017). Increased connectivity of hub networks and cognitive impairment in multiple sclerosis. *Neurology,**88*(22), 2107–2114. 10.1212/WNL.0000000000003982 28468841 10.1212/WNL.0000000000003982

[CR63] Migliore, S., Ghazaryan, A., Simonelli, I., Pasqualetti, P., Squitieri, F., Curcio, G., Landi, D., Palmieri, M., Moffa, F., Filippi, M., & Vernieri, F. (2017). Cognitive impairment in relapsing-remitting multiple sclerosis patients with very mild clinical disability. *Behavioural Neurology,**2017*, 7404289. 10.1155/2017/7404289 28912625 10.1155/2017/7404289 PMC5574272

[CR64] Moccia, M., Lanzillo, R., Palladino, R., Chang, K. C., Costabile, T., Russo, C., De Rosa, A., Carotenuto, A., Sacca, F., Maniscalco, G. T., & Brescia Morra, V. (2016). Cognitive impairment at diagnosis predicts 10-year multiple sclerosis progression. *Multiple Sclerosis,**22*(5), 659–667. 10.1177/1352458515599075 26362896 10.1177/1352458515599075

[CR65] Mortensen, G. L., Theódórsdóttir, Á., Sejbæk, T., & Illes, Z. (2020). Patient attitudes to routine cognitive testing in multiple sclerosis. *Patient preference and adherence,**14*, 693–704. 10.2147/PPA.S245623 32308374 10.2147/PPA.S245623 PMC7135142

[CR66] Niccolai, C., Portaccio, E., Goretti, B., Hakiki, B., Giannini, M., Pastò, L., Righini, I., Falautano, M., Minacapelli, E., Martinelli, V., Incerti, C., Nocentini, U., Fenu, G., Cocco, E., Marrosu, M. G., Garofalo, E., Ambra, F. I., Maddestra, M., Consalvo, M., Viterbo, R. G., Trojano, M., Losignore, N. A., Zimatore, G. B., Pietrolongo, E., Lugaresi, A., Pippolo, L., Roscio, M., Ghezzi, A., Castellano, D., Stecchi, S., & Amato, M. P. (2015). A comparison of the brief international cognitive assessment for multiple sclerosis and the brief repeatable battery in multiple sclerosis patients. *BMC Neurology,**15*(1), 204. 10.1186/s12883-015-0460-8 26472052 10.1186/s12883-015-0460-8 PMC4608308

[CR67] Ouzzani, M., Hammady, H., Fedorowicz, Z., & Elmagarmid, A. (2016). Rayyan — A web and mobile app for systematic reviews. *Systematic Reviews, 5*(210). 10.1186/s13643-016-0384-4 10.1186/s13643-016-0384-4 PMC5139140 27919275

[CR68] Ozkul, C., Guclu-Gunduz, A., Eldemir, K., Apaydin, Y., Yazici, G., & Irkec, C. (2020). Clinical features and physical performance in multiple sclerosis patients with and without cognitive impairment: a cross-sectional study. *International Journal of Rehabilitation Research,**43*(4), 316–323. 10.1097/mrr.0000000000000428 32804701 10.1097/MRR.0000000000000428

[CR69] Parsons, O. A., Stewart, K. D., & Arenberg, D. (1957). Impairment of abstracting ability in multiple sclerosis. *The Journal of Nervous And Mental Disease,**125*(2), 221–225. 10.1097/00005053-195704000-00007 13481718 10.1097/00005053-195704000-00007

[CR70] Patti, F., Amato, M. P., Trojano, M., Bastianello, S., Tola, M. R., Goretti, B., Caniatti, L., Di Monte, E., Ferrazza, P., Brescia Morra, V., Lo Fermo, S., Picconi, O., Luccichenti, G., & COGIMUS Study Group. (2009). Cognitive impairment and its relation with disease measures in mildly disabled patients with relapsing-remitting multiple sclerosis: baseline results from the Cognitive Impairment in Multiple Sclerosis (COGIMUS) study. *Multiple Sclerosis,**15*(7), 779–788. 10.1177/1352458509105544 19542262 10.1177/1352458509105544

[CR71] Planche, V., Gibelin, M., Cregut, D., Pereira, B., & Clavelou, P. (2016). Cognitive impairment in a population-based study of patients with multiple sclerosis: Differences between late relapsing-remitting, secondary progressive and primary progressive multiple sclerosis. *European Journal of Neurology,**23*(2), 282–289. 10.1111/ene.12715 25903918 10.1111/ene.12715

[CR72] Portaccio, E., Goretti, B., Zipoli, V., Nacmias, B., Stromillo, M. L., Bartolozzi, M. L., Siracusa, G., Guidi, L., Federico, A., Sorbi, S., De Stefano, M., & Amato, M. P. (2009a). APOE-ε4 is not associated with cognitive impairment in relapsing-remitting multiple sclerosis. *Multiple Sclerosis,**15*(12), 1489–1494. 10.1177/1352458509348512 19965518 10.1177/1352458509348512

[CR73] Portaccio, E., Goretti, B., Zipoli, V., Siracusa, G., Sorbi, S., & Amato, M. (2009b). A short version of Rao’s Brief Repeatable Battery as a screening tool for cognitive impairment in multiple sclerosis. *The Clinical Neuropsychologist,**23*(2), 268–275. 10.1080/13854040801992815 18609336 10.1080/13854040801992815

[CR74] Prakash, R. S., Snook, E. M., Lewis, J. M., Motl, R. W., & Kramer, A. F. (2008). Cognitive impairments in relapsing-remitting multiple sclerosis: A meta-analysis. *Multiple Sclerosis Journal,**14*(9), 1250–1261. 10.1177/1352458508095004 18701571 10.1177/1352458508095004 PMC2847445

[CR75] Preziosa, P., Rocca, M. A., Pagani, E., Stromillo, M. L., Enzinger, C., Gallo, A., Hulst, H. E., Atzori, M., Pareto, D., Riccitelli, G. C., Copetti, M., De Stefano, N., Fazekas, F., Bisecco, A., Barkhof, F., Yousry, T. A., Arevalo, M. J., Filippi, M., MS Group. (2016). Structural MRI correlates of cognitive impairment in patients with multiple sclerosis: A multicenter study. *Human Brain Mapping,**37*(4), 1627–1644. 10.1002/hbm.23125 26833969 10.1002/hbm.23125 PMC6867484

[CR76] Rao, S. M., Leo, G. J., Bernardin, L., & Unverzagt, F. (1991a). Cognitive dysfunction in multiple sclerosis. I. Frequency, patterns, and prediction. *Neurology,**41*(5), 685–691. 10.1212/wnl.41.5.685 2027484 10.1212/wnl.41.5.685

[CR77] Rao, S. M., Leo, G. J., Ellington, L., Nauertz, T., Bernardin, L., & Unverzagt, F. (1991b). Cognitive dysfunction in multiple sclerosis. II. Impact on employment and social functioning. *Neurology,**41*(5), 692–696. 10.1212/wnl.41.5.692 1823781 10.1212/wnl.41.5.692

[CR78] Rayes, H. A., Tani, C., Kwan, A., Marzouk, S., Colosimo, K., Medina-Rosas, J., Mustafa, A., Su, J., Lambiris, P., Mosca, M., & Touma, Z. (2018). What is the prevalence of cognitive impairment in lupus and which instruments are used to measure it? A systematic review and meta-analysis. *Seminars in Arthritis and Rheumatism,**48*(2), 240–255. 10.1016/j.semarthrit.2018.02.007 29571540 10.1016/j.semarthrit.2018.02.007

[CR79] Rimkus, Cd. M., Junqueira Tde, F., Lyra, K. P., Jackowski, M. P., Machado, M. A., Miotto, E. C., Callegaro, D., Otaduy, M. C., & Leite Cda, C. (2011). Corpus callosum microstructural changes correlate with cognitive dysfunction in early stages of relapsing-remitting multiple sclerosis: Axial and radial diffusivities approach. *Multiple Sclerosis International,**2011*, 304875. 10.1155/2011/304875 22096634 10.1155/2011/304875 PMC3197005

[CR80] Rimkus, C. M., Schoonheim, M. M., Steenwijk, M. D., Vrenken, H., Eijlers, A. J., Killestein, J., Wattjes, M. P., Leite, C. C., Barkhof, F., & Tijms, B. M. (2019). Gray matter networks and cognitive impairment in multiple sclerosis. *Multiple Sclerosis,**25*(3), 382–391. 10.1177/1352458517751650 29320933 10.1177/1352458517751650 PMC6393954

[CR81] Rocca, M. A., Valsasina, P., Hulst, H. E., Abdel-Aziz, K., Enzinger, C., Gallo, A., Pareto, D., Riccitelli, G., Muhlert, N., Ciccarelli, O., Barkhof, F., Fazekas, F., Tedeschi, G., Arevalo, M. J., & Filippi, M. (2014). Functional correlates of cognitive dysfunction in multiple sclerosis: A multicenter fMRI study. *Human Brain Mapping,**35*(12), 5799–5814. 10.1002/hbm.22586 25045065 10.1002/hbm.22586 PMC6869325

[CR82] Ruano, L., Branco, M., Portaccio, E., Goretti, B., Niccolai, C., Patti, F., Chisari, C., Gallo, P., Grossi, P., Ghezzi, A., Roscio, M., Mattioli, F., Stampatori, C., Simone, M., Viterbo, R. G., & Amato, M. P. (2018). Patients with paediatric-onset multiple sclerosis are at higher risk of cognitive impairment in adulthood: An Italian collaborative study. *Multiple Sclerosis Journal,**24*(9), 1234–1242. 10.1177/1352458517717341 28654357 10.1177/1352458517717341

[CR83] Ruano, L., Portaccio, E., Goretti, B., Niccolai, C., Severo, M., Patti, F., Cilia, S., Gallo, P., Grossi, P., Ghezzi, A., Roscio, M., Mattioli, F., Stampatori, C., Trojano, M., Viterbo, R. G., & Amato, M. P. (2017). Age and disability drive cognitive impairment in multiple sclerosis across disease subtypes. *Multiple Sclerosis Journal,**23*(9), 1258–1267. 10.1177/1352458516674367 27738090 10.1177/1352458516674367

[CR84] Sacco, R., Bisecco, A., Corbo, D., Della Corte, M., d’Ambrosio, A., Docimo, R., Gallo, A., Esposito, F., Esposito, S., Cirillo, M., Lavorgna, L., Tedeschi, G., & Bonavita, S. (2015). Cognitive impairment and memory disorders in relapsing-remitting multiple sclerosis: The role of white matter, gray matter and hippocampus. *Journal of Neurology,**262*(7), 1691–1697. 10.1007/s00415-015-7763-y 25957638 10.1007/s00415-015-7763-y

[CR85] Scherer, R. & Emslander, V. (2024). *Utilizing primary study quality in meta-analyses in psychology: A step-by-step tutorial*. PsyArXiv. 10.31234/osf.io/8emsa 10.1037/met0000751 40471813

[CR86] Schoonhoven, D. N., Fraschini, M., Tewarie, P., Uitdehaag, B. M., Eijlers, A. J., Geurts, J. J., Hillebr, A., Schoonheim, M. M., Stam, C. J., & Strijbis, E. M. (2019). Resting-state MEG measurement of functional activation as a biomarker for cognitive decline in MS. *Multiple Sclerosis,**25*(14), 1896–1906. 10.1177/1352458518810260 30465461 10.1177/1352458518810260 PMC6875827

[CR87] Sehanovic, A., Smajlovic, D., Tupkovic, E., Ibrahimagic, O. C., Kunic, S., Dostovic, Z., Zoletic, E., & Pasic, Z. (2020). Cognitive disorders in patients with multiple sclerosis. *Materia Sociomedica, 32*(3), 191. https://simsrad.net.ocs.mq.edu.au/login?url=http://ovidsp.ovid.com/ovidweb.cgi?T=JS&CSC=Y&NEWS=N&PAGE=fulltext&D=pmnm&AN=33424448https://libkey.io/libraries/590/openurl?sid=OVID:medline&id=pmid:33424448&id=doi:10.5455%2Fmsm.2020.32.191-195&issn=1512-7680&isbn=&volume=32&issue=3&spage=191&pages=191-195&date=2020&title=Materia+Sociomedica&atitle=Cognitive+Disorders+in+Patients+with+Multiple+Sclerosis.&aulast=Sehanovic 10.5455/msm.2020.32.191-195 PMC7780783 33424448

[CR88] Sexton, E., McLoughlin, A., Williams, D. J., Merriman, N. A., Donnelly, N., Rohde, D., Hickey, A., Wren, M. A., & Bennett, K. (2019). Systematic review and meta-analysis of the prevalence of cognitive impairment no dementia in the first year post-stroke. *European Stroke Journal,**4*(2), 160–171. 10.1177/2396987318825484 31259264 10.1177/2396987318825484 PMC6591758

[CR89] Singer, B. A., Keith, S., Howerter, A., Doll, H., Pham, T., & Mehta, R. (2021). A study comparing patient and clinician perspectives of treatments for multiple sclerosis via group concept mapping. *Patient Preference and Adherence,**15*, 975–987. 10.2147/ppa.S297052 34012257 10.2147/PPA.S297052 PMC8126969

[CR90] Skorve, E., Lundervold, A. J., Torkildsen, O., & Myhr, K. M. (2023). Assessment of cognitive function in early stages of multiple sclerosis. Validation of BICAMS in Norway. *Multiple Sclerosis Journal,**24*, 798. 10.1016/j.msard.2022.104398 10.1016/j.msard.2022.104398 36462469

[CR91] Strober, L., Englert, J., Munschauer, F., Weinstock-Guttman, B., Rao, S., & Benedict, R. H. (2009). Sensitivity of conventional memory tests in multiple sclerosis: Comparing the Rao Brief Repeatable Neuropsychological Battery and the Minimal Assessment of Cognitive Function in MS. *Multiple Sclerosis,**15*(9), 1077–1084. 10.1177/1352458509106615 19556311 10.1177/1352458509106615

[CR92] Sumowski, J. F., Benedict, R., Enzinger, C., Filippi, M., Geurts, J. J., Hamalainen, P., Hulst, H., Inglese, M., Leavitt, V. M., Rocca, M. A., Rosti-Otajarvi, E. M., & Rao, S. (2018). Cognition in multiple sclerosis: State of the field and priorities for the future. *Neurology,**90*(6), 278–288. 10.1212/wnl.0000000000004977 29343470 10.1212/WNL.0000000000004977 PMC5818015

[CR93] Talebi, M., Sadigh-Eteghad, S., Talebi, M., Naseri, A., & Zafarani, F. (2022). Predominant domains and associated demographic and clinical characteristics in multiple sclerosis-related cognitive impairment in mildly disabled patients. *The Egyptian Journal of Neurology, Psychiatry and Neurosurgery,**58*(1), 48. 10.1186/s41983-022-00485-7

[CR94] Tierney, J. F., Stewart, L. A., & Clarke, M. (2023) Chapter 26: Individual participant data. In: J. P. T. Higgins, J. Thomas, J. Chandler, M. Cumpston, T. Li, M. J. Page, V. A. Welch (Eds.) *Cochrane Handbook for Systematic Reviews of Interventions version 6.4* (updated August 2023). Cochrane, 2023. Available from www.training.cochrane.org/handbook

[CR95] Topcular, B., Ozcan, M., Kurt, E., Kuscu, D., Icen, N., Sutlas, P., Kirbas, D., & Bingol, A. (2012). Cognitive impairment in relapsing-remitting multiple sclerosis. *Noropsikiyatri Arsivi,**49*(3), 178–182. 10.4274/npa.y6089

[CR96] Tran, T., Milanovic, M., Holshausen, K., & Bowie, C. R. (2021). What is normal cognition in depression? Prevalence and functional correlates of normative versus idiographic cognitive impairment. *Neuropsychology,**35*(1), 33. 10.1037/neu0000717 33393798 10.1037/neu0000717

[CR97] Walker, B. F. (2000). The prevalence of low back pain: A systematic review of the literature from 1966 to 1998. *J Spinal Disord,**13*(3), 205–217. 10.1097/00002517-200006000-00003 10872758 10.1097/00002517-200006000-00003

[CR98] Walton, C., King, R., Rechtman, L., Kaye, W., Leray, E., Marrie, R. A., Robertson, N., La Rocca, N., Uitdehaag, B., van der Mei, I., Wallin, M., Helme, A., Angood Napier, C., Rijke, N., & Baneke, P. (2020). Rising prevalence of multiple sclerosis worldwide: Insights from the Atlas of MS, third edition. *Multiple Sclerosis,**26*(14), 1816–1821. 10.1177/1352458520970841 33174475 10.1177/1352458520970841 PMC7720355

[CR99] Whiting, P. F., Rutjes, A. W., Westwood, M. E., Mallett, S., Deeks, J. J., Reitsma, J. B., Leeflang, M. M., Sterne, J. A., & Bossuyt, P. M. (2011). QUADAS-2: A revised tool for the quality assessment of diagnostic accuracy studies. *Annals of Internal Medicine,**155*(8), 529–536. 10.7326/0003-4819-155-8-201110180-00009 22007046 10.7326/0003-4819-155-8-201110180-00009

[CR100] Yazgan, Y. Z., Tarakcı, E., Gungor, F., & Kurtuncu, M. (2021). Understanding the impact of cognitive impairment and disease severity on activities of daily living in MS patients with different disability levels. *Clinical Neurology and Neurosurgery,**200*, 106398. 10.1016/j.clineuro.2020.106398 33310534 10.1016/j.clineuro.2020.106398

[CR101] Yohannes, A. M. P. M. F., Chen, W. P. M. P. H. M., Moga, A. M. M., Leroi, I. M., & Connolly, M. J. M. D. (2017). Cognitive impairment in chronic obstructive pulmonary disease and chronic heart failure: A systematic review and meta-analysis of observational studies. *Journal of the American Medical Directors Association,**18*(5), 451.e451-451.e411. 10.1016/j.jamda.2017.01.014 10.1016/j.jamda.2017.01.014 28292570

[CR102] Van Schependom, J., D’Hooghe, M. B., Cleynhens, K., D’Hooge, M., Haelewyck, M. C., De Keyser, J., & Nagels, G. (2014). The Symbol Digit Modalities Test as sentinel test for cognitive impairment in multiple sclerosis. *European Journal of Neurology,**21*(9), 1219–e72. 10.1111/ene.12463 24850580 10.1111/ene.12463

[CR103] Winter, M., Tallantyre, E. C., Brice, T. A. W., Robertson, N. P., Jones, D. K., & Chamberland, M. (2021). Tract-specific MRI measures explain learning and recall differences in multiple sclerosis. *Brain Communications,**3*(2), fcab065. 10.1093/braincomms/fcab065 33959710 10.1093/braincomms/fcab065 PMC8088789

[CR104] Zhang, X., Zhang, F., Huang, D., Wu, L., Ma, L., Liu, H., Zhao, Y., Yu, S., & Shi, J. (2016). Contribution of gray and white matter abnormalities to cognitive impairment in multiple sclerosis. *International Journal of Molecular Sciences,**18*(1), 27. 10.3390/ijms18010046 28035997 10.3390/ijms18010046 PMC5297681

