# Supplementary material for: The Prevalence of Cognitive Impairment in Relapsing-Remitting Multiple Sclerosis: A Systematic Review and Meta-analysis
Source: Neuropsychol Rev. 2024 Apr 8;35(2):233–53. doi: 10.1007/s11065-024-09640-8 (PMC12328523; doi:10.1007/s11065-024-09640-8)
Supplement: Supplementary file 1 — Supplementary file1 (DOCX 48 kb) [file 11065_2024_9640_MOESM1_ESM.docx]

**SUPPLEMENTARY INFORMATION**

**The Prevalence of Cognitive Impairment in Relapsing-Remitting Multiple Sclerosis:
A Systematic Review and Meta-analysis**

**Figure 1**

Search Strategy

Embase:

1. multiple sclerosis.ab,ti,kw.
2. Mild Cognitive Impairment/or Cognitive Defect/ or
   (cogniti* adj1 (ability or function or impair* or deficit or dysfunction or decline)).ab,ti,kw.
3. Neuropsychological Test/ or Cognition Assessment/ or (neuropsycholog* adj1 (assess* or exam* or test)).ab,ti,kw. or (cogniti* adj1 (assess* or exam* or test)).ab,ti,kw.
4. 1 and (2 or 3)

Medline:

1. multiple sclerosis.mp
2. Cognitive Dysfunction/ or Cognition Disorders/ or Neurocognitive Disorders/ or (cogniti* adj1 (ability or function or impair* or deficit or dysfunction or decline)).mp
3. Neuropsychological Tests/ or Intelligence Tests or (neuropsycholog* adj1 (assess* or exam* or test)).mp or (cogniti* adj1 (assess* or exam* or test)).mp
4. 1 and (2 or 3)

PsycINFO:

1. multiple sclerosis.ab.ti.id
2. Mild Cognitive Impairment/ or Cognitive Impairment/ or Neurocognitive Disorders/ or (cogniti* adj1 (ability or function or impair* or deficit or dysfunction or decline)).ab.ti.id.
3. Neuropsychological Assessment/ or Cognitive Assessment/ or (neuropsycholog* adj1 (assess* or exam* or test)).ab.ti.id. or (cogniti* adj1 (assess* or exam* or test)).ab.ti.id.
4. 1 and (2 or 3)

Scopus:

1. TITLE-ABS-KEY(multiple sclerosis)
2. TITLE-ABS-KEY(cogniti* W/0 (ability OR function OR impair* OR deficit OR dysfunction OR decline))
3. TITLE-ABS-KEY((neuropsycholog* W/0 (assess* OR exam* OR test)) OR (cogniti* W/0 ( assess* OR exam* OR test)))
4. #1 AND (#2 OR #3)

**Table 1**

Study quality assessment using the modified QUADAS.

|  |  | Criterion |
| --- | --- | --- |
| A |  | The final sample should be representative of the target population. |
|  | 1 | At least one of the following should apply for the study (2 points):  An entire target population  Randomly selected sample  Sample stated to represent the target population |
|  | 2 | At least one of the following (2 points):  Reasons for nonresponders described  Nonresponders described  Comparison of responders and nonresponders  Comparison of sample and target population |
|  | 3 | Response rate ≥90% (2 points)  Response rate 70% to 90% (1 point)  Response rate ≤70% (0 point) |
| B |  | Quality of data |
|  | 4 | Were the data primary from a prevalence study (2 points), or was it taken from a survey not specifically designed for that purpose (1 point) |
|  | 5 | The same mode of data collection should be used for all subjects (2 points), if not: 1 point |
|  | 6 | The data have been collected directly from the patient by means of a validated questionnaire/interview (3 points)  No validated questionnaire/interview patient (2 points)  Data have been collected from proxies of retrospectively from  medical record (1 point) |
| C |  | General description of the method and results should include: |
|  | 7 | Description of target population and setting where patients were  found (2 points) |
|  | 8 | Description of stage of disease, sex, age (all 2 points, 1 or 2: 1 point) |
|  | 9 | Final sample size (1 point) |
| D |  | Definitions of prevalence |
|  | 10 | Prevalence recall periods should be stated (1 point) |

**Figure 2**

Reference list of studies included in the review.

Akbar, N., Lobaugh, N. J., O'Connor, P., Moradzadeh, L., Scott, C. J., & Feinstein, A. (2010). Diffusion tensor imaging abnormalities in cognitively impaired multiple sclerosis patients. *Canadian Journal of Neurological Sciences, 37*(5), 608-614. https://doi.org/https://doi.org/10.1017/S0317167100010775

Altieri, M., Fratino, M., Maestrini, I., Dionisi, C., Annecca, R., Vicenzini, E., & Di Piero, V. (2021). Cognitive Performance in Relapsing-Remitting Multiple Sclerosis: At Risk or Impaired? *Dementia and Geriatric Cognitive Disorders, 49*(6), 539-543. https://doi.org/https://doi.org/10.1159/000514674

Amato, M., Bartolozzi, M., Zipoli, V., Portaccio, E., Mortilla, M., Guidi, L., Siracusa, G., Sorbi, S., Federico, A., & De Stefano, N. (2004). Neocortical volume decrease in relapsing-remitting MS patients with mild cognitive impairment. *Neurology, 63*(1), 89-93. https://doi.org/https://doi.org/10.1212/01.wnl.0000129544.79539.d5

Amato, M., Hakiki, B., Goretti, B., Rossi, F., Stromillo, M., Giorgio, A., Roscio, M., Ghezzi, A., Guidi, L., Bartolozzi, M., Portaccio, E., & De Stefano, N. (2012). Association of MRI metrics and cognitive impairment in radiologically isolated syndromes. *Neurology, 78*(5), 309-314. https://doi.org/https://doi.org/10.1212/wnl.0b013e31824528c9

Amato, M., Portaccio, E., Stromillo, M., Goretti, B., Zipoli, V., Siracusa, G., Battaglini, M., Giorgio, A., Bartolozzi, M., Guidi, L., Sorbi, S., Federico, A., & De Stefano, N. (2008). Cognitive assessment and quantitative magnetic resonance metrics can help to identify benign multiple sclerosis. *Neurology, 71*(9), 632-638. https://doi.org/https://doi.org/10.1212/01.wnl.0000324621.58447.00

Amato, M. P., Portaccio, E., Goretti, B., Zipoli, V., Iudice, A., Della Pina, D., Malentacchi, G., Sabatini, S., Annunziata, P., Falcini, M., Mazzoni, M., Mortilla, M., Fonda, C., De Stefano, N., & Tu, S. S. G. (2010). Relevance of cognitive deterioration in early relapsing-remitting MS: a 3-year follow-up study. *Multiple Sclerosis, 16*(12), 1474-1482. https://doi.org/https://doi.org/10.1177/1352458510380089

Bisecco, A., Rocca, M., Pagani, E., Mancini, L., Enzinger, C., Gallo, A., Vrenken, H., Stromillo, M. L., Copetti, M., Thomas, D., Fazekas, F., Tedeschi, G., Barkhof, F., De Stefano, N., & Filippi, M. (2015). Connectivity-based parcellation of the thalamus in multiple sclerosis and its implications for cognitive impairment: A multicenter study. *Human Brain Mapping, 36*(7). https://doi.org/https://doi.org/10.1002/hbm.22809

Caceres, F., Vanotti, S., Benedict, R. H., & Group, R. W. (2014). Cognitive and neuropsychiatric disorders among multiple sclerosis patients from Latin America: Results of the RELACCEM study. *Multiple Sclerosis and Related Disorders, 3*(3), 335-340. https://doi.org/http://dx.doi.org/10.1016/j.msard.2013.10.007

Carotenuto, A., Valsasina, P., Schoonheim, M. M., Geurts, J. J. G., Barkhof, F., Gallo, A., Tedeschi, G., Tommasin, S., Pantano, P., Filippi, M., & Rocca, M. A. (2022). Investigating Functional Network Abnormalities and Associations With Disability in Multiple Sclerosis. *Neurology, 99*(22), e2517-e2530. https://doi.org/https://doi.org/10.1212/wnl.0000000000201264

Conti, L., Preziosa, P., Meani, A., Vizzino, C., Riccitelli, G., Pagani, E., Valsasina, P., Marchesi, O., Filippi, M., & Rocca, M. A. (2021, December). Unraveling the substrates of cognitive impairment in multiple sclerosis: the contribution of a multiparametric structural and functional mri approach. *Multiple Sclerosis Journal, 26*, 513. https://doi.org/https://doi.org/10.1111/ene.15023

d'Ambrosio, A., Valsasina, P., Gallo, A., De Stefano, N., Pareto, D., Barkhof, F., Ciccarelli, O., Enzinger, C., Tedeschi, G., Stromillo, M. L., Arévalo, M. J., Hulst, H. E., Muhlert, N., Koini, M., Filippi, M., & Rocca, M. A. (2020). Reduced dynamics of functional connectivity and cognitive impairment in multiple sclerosis. *Mult Scler, 26*(4), 476-488. https://doi.org/https://doi.org/10.1177/1352458519837707

Damasceno, A., Pimentel-Silva, L. R., Damasceno, B. P., & Cendes, F. (2020). Cognitive trajectories in relapsing–remitting multiple sclerosis: A longitudinal 6-year study. *Multiple Sclerosis Journal, 26*(13), 1740-1751. https://doi.org/https://doi.org/10.1177/1352458519878685

Deloire, M. S., Salort, E., Bonnet, M., Arimone, Y., Boudineau, M., Amieva, H., Barroso, B., Ouallet, J. C., Pachai, C., Galliaud, E., Petry, K. G., Dousset, V., Fabrigoule, C., & Brochet, B. (2005). Cognitive impairment as marker of diffuse brain abnormalities in early relapsing remitting multiple sclerosis. *Journal of Neurology, Neurosurgery & Psychiatry, 76*(4), 519-526. https://doi.org/https://doi.org/10.1136/jnnp.2004.045872

Dusankova, J. B., Kalincik, T., Havrdova, E., & Benedict, R. H. B. (2012). Cross cultural validation of the minimal assessment of cognitive function in multiple sclerosis (MACFIMS) and the brief international cognitive assessment for multiple sclerosis (BICAMS). *Clinical Neuropsychologist, 26*(7), 1186-1200. https://doi.org/https://doi.org/10.1080/13854046.2012.725101

Eshaghi, A., Riyahi-Alam, S., Roostaei, T., Haeri, G., Aghsaei, A., Aidi, M. R., Pouretemad, H. R., Zarei, M., Farhang, S., Saeedi, R., Nazeri, A., Ganjgahi, H., Etesam, F., Azimi, A. R., Benedict, R. H. B., & Sahraian, M. A. (2012). Validity and reliability of a Persian translation of the Minimal Assessment of Cognitive Function in Multiple Sclerosis (MACFIMS) [Article]. *Clinical Neuropsychologist, 26*(6), 975-984. https://doi.org/https://doi.org/10.1080/13854046.2012.694912

Gajofatto, A., Turatti, M., Bianchi, M., Forlivesi, S., Gobbin, F., Azzara, A., Monaco, S., & Benedetti, M. (2016). Benign multiple sclerosis: Physical and cognitive impairment follow distinct evolutions. *Acta Neurologica Scandinavica, 133*(3), 183-191. https://doi.org/https://doi.org/10.1111/ane.12442

Gois, L. C. P., Pimentel-Silva, L. R., Damasceno, B. P., & Damasceno, A. (2021). Associations between cognitive and clinical disability across MS subtypes: The role of the underlying brain damage. *Multiple Sclerosis and Related Disorders, 48*, 102701. https://doi.org/https://doi.org/10.1016/j.msard.2020.102701

Goretti, B., Viterbo, R., Portaccio, E., Niccolai, C., Hakiki, B., Piscolla, E., Iaffaldano, P., Trojano, M., & Amato, M. (2014). Anxiety state affects information processing speed in patients with multiple sclerosis. *Neurological Sciences, 35*(4), 559-563. https://doi.org/https://doi.org/10.1007/s10072-013-1544-0

Hulst, H. E., Schoonheim, M. M., Roosendaal, S. D., Popescu, V., Schweren, L. J., van der Werf, Y. D., Visser, L. H., Polman, C. H., Barkhof, F., & Geurts, J. J. (2012). Functional adaptive changes within the hippocampal memory system of patients with multiple sclerosis. *Hum Brain Mapp, 33*(10), 2268-2280. https://doi.org/https://doi.org/10.1002/hbm.21359

Iaffaldano, P., Viterbo, R. G., Goretti, B., Portaccio, E., Amato, M. P., & Trojano, M. (2014). Emotional and neutral verbal memory impairment in multiple sclerosis. *Multiple Sclerosis, 1*, 408-409. https://doi.org/http://dx.doi.org/10.1016/j.jns.2014.03.038

Jakimovski, D., Weinstock-Guttman, B., Roy, S., Jaworski, M., Hancock, L., Nizinski, A., Srinivasan, P., Fuchs, T. A., Szigeti, K., Zivadinov, R., & Benedict, R. H. (2019). Cognitive profiles of aging in multiple sclerosis. *Frontiers in Aging Neuroscience, 11*. https://doi.org/https://doi.org/10.3389/fnagi.2019.00105

Jandric, D., Lipp, I., Paling, D., Rog, D., Castellazzi, G., Haroon, H., Parkes, L., Parker, G., Tomassini, V., & Muhlert, N. (2021). Mechanisms of network changes in cognitive impairment in multiple sclerosis. *Neurology, 97*, 19. https://doi.org/https://doi.org/10.1212/WNL.0000000000012834

Jonkman, L., Rosenthal, D. M., Sormani, M. P., Miles, L., Herbert, J., Grossman, R. I., & Inglese, M. (2015). Gray matter correlates of cognitive performance differ between relapsing-remitting and primary-progressive multiple sclerosis. *Multiple Sclerosis, 1*, 362. https://doi.org/https://doi.org/10.1371/journal.pone.0129380

Lanzillo, R., Prinster, A., Scarano, V., Liuzzi, R., Coppola, G., Florio, C., Salvatore, E., Schiavone, V., Brunetti, A., Muto, M., Orefice, G., Alfano, B., Bonavita, V., & Morra, V. B. (2006). Neuropsychological assessment, quantitative MRI and ApoE gene polymorphisms in a series of MS patients treated with IFN beta-1b. *Journal of the Neurological Sciences, 245*(1-2), 141-145. https://doi.org/https://doi.org/10.1016/j.jns.2005.08.023

Lozano-Soto, E., Cruz-López, Á., Gutiérrez, R., González, M., Sanmartino, F., Rashid-Lopez, R., Espinosa-Rosso, R., Forero, L., & González-Rosa, J. J. (2021). Predicting Neuropsychological Impairment in Relapsing Remitting Multiple Sclerosis: The Role of Clinical Measures, Treatment, and Neuropsychiatry Symptoms. *Archives of clinical neuropsychology : the official journal of the National Academy of Neuropsychologists, 36*(4), 475-484. https://doi.org/https://doi.org/10.1093/arclin/acaa088

Ma, A. Y., Vitorino, R. C., Hojjat, S. P., Mulholl, , A. D., Zhang, L., Lee, L., Carroll, T. J., Cantrell, C. G., Figley, C. R., & Aviv, R. I. (2017). The relationship between white matter fiber damage and gray matter perfusion in largescale functionally defined networks in multiple sclerosis. *Multiple Sclerosis Journal, 23*, 1884-1892. https://doi.org/https://doi.org/10.1177/1352458517691149

Maarouf, A., Audoin, B., Pariollaud, F., Gherib, S., Rico, A., Soulier, E., Confort-Gouny, S., Guye, M., Schad, L., Pelletier, J., Ranjeva, J.-P., & Zaaraoui, W. (2017). Increased total sodium concentration in gray matter better explains cognition than atrophy in MS. *Neurology, 88*(3), 289-295. https://doi.org/https://doi.org/10.1212/WNL.0000000000003511

Mashayekhi, F., Sadigh-Eteghad, S., Naseri, A., Asadi, M., Abbasi Garravnd, N., & Talebi, M. (2022). ApoE4-positive multiple sclerosis patients are more likely to have cognitive impairment: a cross-sectional study. *Neurol Sci, 43*(2), 1189-1196. https://doi.org/https://doi,org/10.1007/s10072-021-05383-z

Maubeuge, N., Deloire, M. S. A., Brochet, B., Ehrle, N., Charre-Morin, J., Saubusse, A., Ruet, A., & investigators, B. s. (2021). Validation of the French version of the minimal assessment of cognitive function in multiple sclerosis (MACFIMS). *Multiple Sclerosis and Related Disorders, 48*, 102692. https://doi.org/https://doi.org/10.1016/j.msard.2020.102692

Meijer, K. A., Eijlers, A. J. C., Douw, L., Uitdehaag, B. M., Barkhof, F., Geurts, J. J., & Schoonheim, M. M. (2017). Increased connectivity of hub networks and cognitive impairment in multiple sclerosis. *Neurology, 88*(22), 2107-2114. https://doi.org/https://doi.org/10.1212/WNL.0000000000003982

Migliore, S., Ghazaryan, A., Simonelli, I., Pasqualetti, P., Squitieri, F., Curcio, G., i, D., Palmieri, M., Moffa, F., Filippi, M., & Vernieri, F. (2017, Aug 15). Cognitive impairment in relapsing-remitting multiple sclerosis patients with very mild clinical disability. *Behavioural Neurology 2017*, ArtID 7404289. https://doi.org/https://doi.org/10.1155/2017/7404289

Moccia, M., Lanzillo, R., Palladino, R., Chang, K. C., Costabile, T., Russo, C., De Rosa, A., Carotenuto, A., Sacca, F., Maniscalco, G. T., & Brescia Morra, V. (2016). Cognitive impairment at diagnosis predicts 10-year multiple sclerosis progression. *Multiple Sclerosis, 22*(5), 659-667. https://doi.org/ https://doi.org/10.1177/1352458515599075

Niccolai, C., Portaccio, E., Goretti, B., Hakiki, B., Giannini, M., Pastò, L., Righini, I., Falautano, M., Minacapelli, E., Martinelli, V., Incerti, C., Nocentini, U., Fenu, G., Cocco, E., Marrosu, M. G., Garofalo, E., Ambra, F. I., Maddestra, M., Consalvo, M., Viterbo, R. G., Trojano, M., Losignore, N. A., Zimatore, G. B., Pietrolongo, E., Lugaresi, A., Pippolo, L., Roscio, M., Ghezzi, A., Castellano, D., Stecchi, S., & Amato, M. P. (2015). A comparison of the brief international cognitive assessment for multiple sclerosis and the brief repeatable battery in multiple sclerosis patients. *BMC Neurology, 15*(1), 204-204. https://doi.org/https://doi.org/10.1186/s12883-015-0460-8

Ozkul, C., Guclu-Gunduz, A., Eldemir, K., Apaydin, Y., Yazici, G., & Irkec, C. (2020). Clinical features and physical performance in multiple sclerosis patients with and without cognitive impairment: a cross-sectional study. *Int J Rehabil Res, 43*(4), 316-323. https://doi.org/https://doi.org/10.1097/mrr.0000000000000428

Patti, F., Amato, M. P., Trojano, M., Bastianello, S., Tola, M. R., Goretti, B., Caniatti, L., Di Monte, E., Ferrazza, P., Brescia Morra, V., Lo Fermo, S., Picconi, O., Luccichenti, G., & Group, C. S. (2009). Cognitive impairment and its relation with disease measures in mildly disabled patients with relapsing-remitting multiple sclerosis: baseline results from the Cognitive Impairment in Multiple Sclerosis (COGIMUS) study. *Multiple Sclerosis, 15*(7), 779-788. https://doi.org/https://doi.org/10.1177/1352458509105544

Portaccio, E., Goretti, B., Zipoli, V., Nacmias, B., Stromillo, M. L., Bartolozzi, M. L., Siracusa, G., Guidi, L., Federico, A., Sorbi, S., De Stefano, M., & Amato, M. P. (2009). APOE-ε4 is not associated with cognitive impairment in relapsing-remitting multiple sclerosis. *Multiple Sclerosis, 15*(12), 1489-1494. https://doi.org/https://doi.org/10.1177/1352458509348512

Portaccio, E., Goretti, B., Zipoli, V., Siracusa, G., Sorbi, S., & Amato, M. (2009). A short version of Rao's Brief Repeatable Battery as a screening tool for cognitive impairment in multiple sclerosis. *The Clinical Neuropsychologist, 23*(2), 268-275. https://doi.org/https://doi.org/10.1080/13854040801992815

Preziosa, P., Rocca, M. A., Pagani, E., Stromillo, M. L., Enzinger, C., Gallo, A., Hulst, H. E., Atzori, M., Pareto, D., Riccitelli, G. C., Copetti, M., De Stefano, N., Fazekas, F., Bisecco, A., Barkhof, F., Yousry, T. A., Arevalo, M. J., Filippi, M., & Group, M. S. (2016). Structural MRI correlates of cognitive impairment in patients with multiple sclerosis: A Multicenter Study. *Human Brain Mapping, 37*(4), 1627-1644. https://doi.org/https://doi.org/10.1002/hbm.23125

Rimkus, C. d. M., Junqueira Tde, F., Lyra, K. P., Jackowski, M. P., Machado, M. A., Miotto, E. C., Callegaro, D., Otaduy, M. C., & Leite Cda, C. (2011). Corpus callosum microstructural changes correlate with cognitive dysfunction in early stages of relapsing-remitting multiple sclerosis: Axial and radial diffusivities approach. *Multiple Sclerosis International, 2011*, 304875. https://doi.org/https://doi.org/10.1155/2011/304875

Rimkus, C. M., Schoonheim, M. M., Steenwijk, M. D., Vrenken, H., Eijlers, A. J., Killestein, J., Wattjes, M. P., Leite, C. C., Barkhof, F., & Tijms, B. M. (2019). Gray matter networks and cognitive impairment in multiple sclerosis. *Multiple Sclerosis, 25*(3), 382-391. https://doi.org/https://doi.org/10.1177/1352458517751650

Rocca, M. A., Valsasina, P., Hulst, H. E., Abdel-Aziz, K., Enzinger, C., Gallo, A., Pareto, D., Riccitelli, G., Muhlert, N., Ciccarelli, O., Barkhof, F., Fazekas, F., Tedeschi, G., Arevalo, M. J., & Filippi, M. (2014). Functional correlates of cognitive dysfunction in multiple sclerosis: A multicenter fMRI study. *Human Brain Mapping, 35*(12), 5799-5814. https://doi.org/https://doi.org/10.1002/hbm.22586

Ruano, L., Portaccio, E., Goretti, B., Niccolai, C., Severo, M., Patti, F., Cilia, S., Gallo, P., Grossi, P., Ghezzi, A., Roscio, M., Mattioli, F., Stampatori, C., Trojano, M., Viterbo, R. G., & Amato, M. P. (2017). Age and disability drive cognitive impairment in multiple sclerosis across disease subtypes. *Multiple Sclerosis Journal, 23*(9), 1258-1267. https://doi.org/https://doi.org/10.1177/1352458516674367

Sacco, R., Bisecco, A., Corbo, D., Della Corte, M., d’Ambrosio, A., Docimo, R., Gallo, A., Esposito, F., Esposito, S., Cirillo, M., Lavorgna, L., Tedeschi, G., & Bonavita, S. (2015). Cognitive impairment and memory disorders in relapsing–remitting multiple sclerosis: the role of white matter, gray matter and hippocampus. *Journal of Neurology, 262*(7), 1691-1697. https://doi.org/https://doi.org/10.1007/s00415-015-7763-y

Schoonhoven, D. N., Fraschini, M., Tewarie, P., Uitdehaag, B. M., Eijlers, A. J., Geurts, J. J., Hillebr, A., Schoonheim, M. M., Stam, C. J., & Strijbis, E. M. (2019). Resting-state MEG measurement of functional activation as a biomarker for cognitive decline in MS. *Multiple Sclerosis, 25*(14), 1896-1906. https://doi.org/https://doi.org/10.1177%2F1352458518810260

Skorve, E., Lundervold, A. J., Torkildsen, O., & Myhr, K. M. (2023). Assessment of cognitive function in early stages of multiple sclerosis. Validation of BICAMS in Norway. *Multiple Sclerosis Journal, 24*, 798. https://doi.org/https://doi.org/10.1016/j.msard.2022.104398

Talebi, M., Sadigh-Eteghad, S., Talebi, M., Naseri, A., & Zafarani, F. (2022). Predominant domains and associated demographic and clinical characteristics in multiple sclerosis-related cognitive impairment in mildly disabled patients. *The Egyptian Journal of Neurology, Psychiatry and Neurosurgery, 58*(1), 48. https://doi.org/https://doi.org/10.1186/s41983-022-00485-7

Topcular, B., Ozcan, M., Kurt, E., Kuscu, D. Y., im, Icen, N. K., Sutlas, P. N., Kirbas, D., & Bingol, A. (2012). Cognitive impairment in relapsing-remitting multiple sclerosis. *Noropsikiyatri Arsivi, 49*(3), 178-182. https://doi.org/https://doi.org/10.4274/npa.y6089

Van Schependom, J., D'Hooghe, M. B., Cleynhens, K., D'Hooge, M., Haelewyck, M. C., De Keyser, J., & Nagels, G. (2014). The Symbol Digit Modalities Test as sentinel test for cognitive impairment in multiple sclerosis. *European Journal of Neurology, 21*(9), 1219-1211e1272. https://doi.org/https://doi.org/10.1111/ene.12463

Winter, M., Tallantyre, E. C., Brice, T. A. W., Robertson, N. P., Jones, D. K., Chamberl, & , M. (2021). Tract-specific MRI measures explain learning and recall differences in multiple sclerosis. *Brain Communications, 3*(2), fcab065. https://doi.org/https://doi.org/10.1093/braincomms/fcab065

Zhang, X., Zhang, F., Huang, D., Wu, L., Ma, L., Liu, H., Zhao, Y., Yu, S., & Shi, J. (2016). Contribution of Gray and White Matter Abnormalities to Cognitive Impairment in Multiple Sclerosis. *International Journal of Molecular Sciences, 18*(1), 27. https://doi.org/https://doi.org/10.3390/ijms18010046

**Figure 3**

Funnel Plot

**Table 2**

Study Quality Ratings

| **Study** | **Criteria (modified QUADAS tool)** | | | | | | | | | | **Total** |
| --- | --- | --- | --- | --- | --- | --- | --- | --- | --- | --- | --- |
|  | 1 | 2 | 3 | 4 | 5 | 6 | 7 | 8 | 9 | 10 |  |
| Akbar et al. (2010) | 0 | 0 | 0 | 2 | 2 | 3 | 2 | 2 | 1 | N/A | 12 |
| Altieri et al. (2021) | 0 | 0 | 0 | 2 | 2 | 3 | 2 | 2 | 1 | N/A | 12 |
| Amato et al. (2004) | 0 | 0 | 0 | 2 | 2 | 3 | 2 | 2 | 1 | N/A | 12 |
| Amato et al. (2008) | 0 | 0 | 0 | 2 | 2 | 3 | 2 | 2 | 1 | N/A | 12 |
| Amato et al. (2010) | 0 | 0 | 0 | 2 | 2 | 3 | 2 | 2 | 1 | N/A | 12 |
| Amato et al. (2012) | 0 | 0 | 0 | 2 | 2 | 3 | 2 | 2 | 1 | N/A | 12 |
| Bisecco et al. (2015) | 0 | 0 | 0 | 2 | 2 | 3 | 2 | 2 | 1 | N/A | 12 |
| Caceres et al. (2014) | 0 | 0 | 0 | 2 | 2 | 3 | 2 | 2 | 1 | N/A | 12 |
| Carotenuto et al. (2022) | 0 | 0 | 0 | 2 | 2 | 3 | 2 | 2 | 1 | N/A | 12 |
| Conti et al. (2021) | 0 | 0 | 0 | 2 | 2 | 3 | 0 | 2 | 1 | N/A | 10 |
| d’Ambrosio et al. (2020) | 0 | 0 | 0 | 2 | 2 | 3 | 2 | 2 | 1 | N/A | 12 |
| Damasceno et al. (2020) | 0 | 0 | 0 | 2 | 2 | 3 | 2 | 2 | 1 | N/A | 12 |
| Deloire et al. (2005) | 0 | 0 | 0 | 2 | 2 | 3 | 2 | 2 | 1 | N/A | 12 |
| Dusankova et al. (2012) | 0 | 0 | 0 | 2 | 2 | 3 | 2 | 2 | 1 | N/A | 12 |
| Eshaghi et al. (2012) | 0 | 0 | 0 | 2 | 2 | 3 | 2 | 2 | 1 | N/A | 12 |
| Gajofatto et al. (2016) | 0 | 0 | 0 | 2 | 2 | 3 | 2 | 2 | 1 | N/A | 12 |
| Gois et al. (2021) | 0 | 0 | 0 | 2 | 2 | 3 | 2 | 2 | 1 | N/A | 12 |
| Goretti et al. (2014) | 0 | 0 | 0 | 2 | 2 | 3 | 2 | 2 | 1 | N/A | 12 |
| Hulst et al. (2012) | 0 | 0 | 0 | 2 | 2 | 3 | 2 | 2 | 1 | N/A | 12 |
| Iaffaldano (2014) | 0 | 0 | 0 | 2 | 2 | 3 | 2 | 2 | 1 | N/A | 12 |
| Jakimovski et al. (2019) | 0 | 0 | 0 | 2 | 2 | 3 | 0 | 2 | 1 | N/A | 10 |
| Jandric et al. (2021) | 0 | 0 | 0 | 2 | 2 | 3 | 2 | 2 | 1 | N/A | 12 |
| Jonkman et al. (2015) | 0 | 0 | 0 | 2 | 2 | 3 | 0 | 2 | 1 | N/A | 10 |
| Lanzillo et al. (2006) | 0 | 0 | 0 | 2 | 2 | 3 | 0 | 2 | 1 | N/A | 10 |
| Lozano-Soto et al. (2021) | 0 | 0 | 0 | 1 | 2 | 3 | 2 | 2 | 1 | N/A | 11 |
| Ma et al. (2017) | 0 | 0 | 0 | 2 | 2 | 3 | 2 | 2 | 1 | N/A | 12 |
| Maarouf et al. (2017) | 0 | 0 | 0 | 2 | 2 | 3 | 2 | 2 | 1 | N/A | 12 |
| Mashayekhi et al. (2021) | 0 | 0 | 0 | 2 | 2 | 3 | 0 | 2 | 1 | N/A | 10 |
| Maubeuge et al. (2021) | 0 | 0 | 0 | 2 | 2 | 3 | 2 | 2 | 1 | N/A | 12 |
| Meijer et al. (2017) | 0 | 0 | 0 | 2 | 2 | 3 | 2 | 2 | 1 | N/A | 12 |
| Migliore et al. (2017) | 2 | 0 | 0 | 2 | 2 | 3 | 2 | 2 | 1 | N/A | 14 |
| Moccia et al. (2016) | 0 | 0 | 0 | 1 | 2 | 3 | 2 | 2 | 1 | N/A | 11 |
| Niccolai et al. (2015) | 0 | 0 | 0 | 2 | 2 | 3 | 2 | 2 | 1 | N/A | 12 |
| Ozkul et al. (2009) | 0 | 0 | 0 | 2 | 2 | 3 | 2 | 2 | 1 | N/A | 12 |
| Patti et al. (2009) | 0 | 0 | 1 | 2 | 2 | 3 | 2 | 2 | 1 | N/A | 13 |
| Portaccio et al. (2009a) | 0 | 0 | 0 | 2 | 2 | 3 | 2 | 2 | 1 | N/A | 12 |
| Portaccio et al. (2009b) | 0 | 0 | 0 | 2 | 2 | 3 | 2 | 2 | 1 | N/A | 12 |
| Preziosa et al. (2016) | 0 | 0 | 0 | 2 | 2 | 3 | 2 | 2 | 1 | N/A | 12 |
| Rimkus et al. (2011) | 0 | 0 | 0 | 2 | 2 | 3 | 2 | 2 | 1 | N/A | 12 |
| Rimkus et al. (2019) | 0 | 0 | 0 | 2 | 2 | 3 | 2 | 2 | 1 | N/A | 12 |
| Rocca et al. (2014) | 0 | 0 | 0 | 2 | 2 | 3 | 2 | 2 | 1 | N/A | 12 |
| Ruano et al. (2017) | 0 | 0 | 1 | 2 | 2 | 3 | 2 | 2 | 1 | N/A | 13 |
| Sacco et al. (2015) | 0 | 0 | 0 | 2 | 2 | 3 | 2 | 2 | 1 | N/A | 12 |
| Schoonhoven et al. (2019) | 0 | 0 | 0 | 1 | 2 | 3 | 2 | 2 | 1 | N/A | 11 |
| Skorve et al. (2023) | 0 | 0 | 0 | 2 | 2 | 3 | 0 | 2 | 1 | N/A | 10 |
| Talebi et al. (2022) | 0 | 0 | 0 | 2 | 2 | 3 | 2 | 2 | 1 | N/A | 12 |
| Topcular et al. (2012) | 0 | 0 | 0 | 2 | 2 | 3 | 2 | 2 | 1 | N/A | 12 |
| Van Schependom et al. (2014) | 0 | 0 | 0 | 1 | 2 | 3 | 2 | 2 | 1 | N/A | 11 |
| Winter et al. (2021) | 0 | 2 | 0 | 2 | 2 | 3 | 0 | 2 | 1 | N/A | 12 |
| Zhang et al. (2016) | 0 | 0 | 0 | 2 | 2 | 3 | 2 | 2 | 1 | N/A | 12 |
